# Supplementary material for: Improving the annotation of the Heterorhabditis bacteriophora genome
Source: Gigascience. 2018 Apr 2;7(4):giy034. doi: 10.1093/gigascience/giy034 (PMC5906903; doi:10.1093/gigascience/giy034)
Supplement: GIGA-D-17-00297_Revision_1.pdf [file giy034_giga-d-17-00297_revision_1.pdf]

GigaScience
Improving the annotation of the Heterorhabditis bacteriophora genome
--Manuscript Draft--

**Manuscript Number:**	GIGA-D-17-00297R1
**Full Title:**	Improving the annotation of the Heterorhabditis bacteriophora genome
**Article Type:**	Data Note
**Funding Information:**	Wellcome Trust (204052/Z/16/Z) — Dr Florence McLean
**Abstract:**	Genome assembly and annotation remains an exacting task. As the tools available for these tasks improve, it is useful to return to data produced with earlier instances to assess their credibility and correctness. The entomopathogenic nematode Heterorhabditis bacteriophora is widely used to control insect pests in horticulture. The genome sequence for this species was reported to encode an unusually high proportion of unique proteins and a paucity of secreted proteins compared to other related nematodes. We revisited the H. bacteriophora genome assembly and gene predictions to ask whether these unusual characteristics were biological or methodological in origin. We mapped an independent resequencing dataset to the genome and used the blobtools pipeline to identify potential contaminants. While present (0.2% of the genome span, 0.4% of predicted proteins), assembly contamination was not significant. Re-prediction of the gene set using BRAKER1 and published transcriptome data generated a predicted proteome that was very different from the published one. The new gene set had a much reduced complement of unique proteins, better completeness values that were in line with other related species' genomes, and an increased number of proteins predicted to be secreted. It is thus likely that methodological issues drove the apparent uniqueness of the initial H. bacteriophora genome annotation and that similar contamination and misannotation issues affect other published genome assemblies.
**Corresponding Author:**	Florence McLean, BM BCh, MSc(R)<br>University of Edinburgh<br>Edinburgh, UNITED KINGDOM
**Corresponding Author Secondary Information:**	
**Corresponding Author's Institution:**	University of Edinburgh
**Corresponding Author's Secondary Institution:**	
**First Author:**	Florence McLean
**First Author Secondary Information:**	
**Order of Authors:**	Florence McLean<br>Duncan Berger<br>Dominik R Laetsch<br>Hillel T Schwartz<br>Mark Blaxter
**Order of Authors Secondary Information:**	
**Response to Reviewers:**	Date 05.03.2018<br><br>Dear GigaScience Editors,<br><br>Re: Resubmission of manuscript: Improving the annotation of the Heterorhabditis bacteriophora genome<br><br>Thank you for the opportunity to revise our manuscript, Improving the annotation of the

Powered by Editorial Manager® and ProduXion Manager® from Aries Systems Corporation

Heterorhabditis bacteriophora genome. After reviewing the GigaScience Article Types instructions we are re-submitting the manuscript for consideration for publication as a Data Note as we agree that it fits best as such.

We are grateful for the constructive and positive suggestions from all of the reviewers, as well as their attention to detail. In particular, we have added a supplementary document detailing the command lines used to carry out the analysis, and hope that this will prove useful to those wishing to replicate the experiments.

Below are the comments with our responses indicated. The accompanying manuscript has the corresponding corrections and changes.

Yours Sincerely,

Florence McLean

Reviewer #1:

This manuscript describes the reannotation of the Heterorhabditis bacteriophora, an entomopathogenic nematode widely used to control insect pests in horticulture.
A previous study was reported to encode an unusually high proportion of unique proteins and a paucity of secreted proteins compared to other related nematodes. This study asked whether these unusual characteristics were biological or methodological in origin.

The work was carried out in the spirit of data improvement, rather than a rebuttal, and while it is not a genome paper as such, it does reanalyse a genome using new data and different tools. It is very suited to the GigaScience philosophy and readership due to the repeatable side and open access component.

I have checked that the Methods described and the Resources used meet the minimum standards reporting check list. I note that data has been submitted to the publicly available repositories (SRA and INSDC) but that the data is not yet available, thus it cannot be reviewed at the moment.

**********Response*******************
The reads from the re-sequencing project are still in the process of being submitted to the SRA and the DOI will be advised as soon as it is obtained.

Submission of the revised annotations to INSDC has been delayed over a question of where they would fit into the ENA's data structure. The GFF file has therefore been submitted to Zenodo (DOI:10.5281/zenodo.1169646), and is included in the supplementary data uploaded to the GigaScience DB.
****

I have looked at the files in https://github.com/DRL/mclean2017
There are 9 supplementary files of annotation, analyses and annotation pipelines which look thorough and complete.

The repository also include splice site files.

The manuscript states that all custom scripts developed for this manuscript are available at in this repository but I see only a single script in the /analysis folder. Is this right?

**********Response*******************
Very few custom scripts were developed for the analysis of the data, the bulk of which was carried out by executing published programs on the command line, and most basic statistics reported in the manuscript (such as counts) were obtained from manipulation and interrogation of files using unix command line tools. Although these processes were not developed as scripts, we strongly agree with both reviewer #1 here, and reviewer #3 (see below), that provision of the code used in the analysis would greatly enhance the manuscript. We have added a Methods Supplementary

Powered by Editorial Manager® and ProduXion Manager® from Aries Systems Corporation

Note (Supplementary File 2) to this effect.
****

The gene prediction and protein orthology analyses and discussion were thorough and fully explained, as well as future work (expanded transcriptome and comparative data work) described.

My recommendation is that this manuscript be published as a research article.

I have some minor typos and suggestions which are probably more pertinent for a copy editor to spot but include them here since I noted them down.

105 BUSCO; see below). Another unusual feature of the H. bacteriophora gene set was the ->
105 BUSCO; see Table 2). Another unusual feature of the H. bacteriophora gene set was the

**********Response*******************
Corrected
****

107 Most nematode (and other metazoan) genomes have low proportions of non-canonical introns (less than 1%),
[Reference needed]

**********Response*******************
Reference provided
****

137 from the new Illumina data and sequence similarity from the NCBI nucleotide database (nt) ->
137 from the new Illumina data and sequence similarity from the NCBI nucleotide (nt) database

**********Response*******************
Corrected
****

371 The assembly scaffolds were aligned to the NCBI nucleotide (nt) database, ->
371 The assembly scaffolds were aligned to the NCBI nt database,

**********Response*******************
Corrected
****

397 version of the assembly. Hard masking was for known Nematoda repeats from the ->
397 version of the assembly. The assembly was hard-masked for known Nematoda repeats from the….?

**********Response*******************
Corrected
****

[Hard masked / hard-masked
Soft masked / soft-masked
check for consistent use]

**********Response*******************
Corrected to consistent use of hyphen
****

406 bacteriophora annotation was identified from the general feature format file, and

Powered by Editorial Manager® and ProduXion Manager® from Aries Systems Corporation

	then-><br>406 bacteriophora annotation was identified from the general feature format (GFF) file, and then<br><br>**********Response*******************<br>Corrected<br>**************************************<br><br>407 selected from the protein FASTA files. The general feature format file (GFF) for -><br>407 selected from the protein FASTA files. The GFF file for<br><br>**********Response*******************<br>Corrected<br>**************************************<br><br>415 from the general feature format file as exon features -><br>415 from the GFF file as exon features<br><br>**********Response*******************<br>Corrected<br>**************************************<br><br>423 bacteriophora. Intronic features were added to GFF3<br>[Explain what GFF3 is]<br><br>Expanded to general feature format version 3 (GFF3)<br><br>[Check consistent use of GFF (line 415) / GFF file / GFF format (744, 749)<br>Should be GFF file]<br><br>**********Response*******************<br>Corrected to be consistent<br>**************************************<br><br>424 gff3 -sort -tidy -retainids -fixregionboundaries -addintrons') and and splice sites were -><br>424 gff3 -sort -tidy -retainids -fixregionboundaries -addintrons') and splice sites were<br><br>**********Response*******************<br>Corrected<br>**************************************<br><br>445 the 23 Clade V nematodes were downloaded from WBPS8 (available at:<br>446 http://parasite.wormbase.org/index.html)<br>[Suggest link to ftp://ftp.ebi.ac.uk/pub/databases/wormbase/parasite/releases/WBPS8/)<br><br>**********Response*******************<br>Link changed to that suggested<br>**************************************<br><br>358 Parasite (WBPS8) [34].<br>[This is the first mention of WormBase Parasite so should include the home page rather than line in 446]<br><br>**********Response*******************<br>Suggested link inserted into formerly line [358] and removed from formerly line [446]<br>**************************************<br><br>478 using MAFFT v7.267 (RRID:SCR_011811) [50], and the alignments trimmed with NOISY<br>[Reference needed for NOISY.]<br><br>**********Response*******************<br>Reference added

Powered by Editorial Manager® and ProduXion Manager® from Aries Systems Corporation

****

480 v8.1.20 (RRID:SCR_006086) [51] with a PROTGAMMAGTR
[Reference needed for PROTGAMMAGTR]

**********Response*******************
Reference provided for RAXML. PROTGAMMAGTR is an option used within RAXML.
****

Reviewer #2:

The manuscript "Improving the annotation of the Heterorhabditis bacteriophora genome" presents the re-annotation of an existing high-quality genome assembly which previously had low-quality gene annotation with many issues. By utilizing RNA-Seq datasets and using the latest high-quality annotation tool (BRAKER1), significant improvements were made in completeness, unique protein counts and secretion predictions. This annotation improvement represents a very significant improvement in how results from Heterorhabditis bacteriophora genome studies will be interpreted.

- The supporting data files are thorough and complete, and support the findings. One suggestion: Although not part of the study, a text file could be added within Supp Tables 2 and 3 which provides the WormBase assembly version used, and accession IDs / web links to the genome assembly, so that readers can have all the information they need to work with the new annotation within the single files.

**********Response*******************
Many thanks for your suggestion. I have added a text file into the Supporting data called Publicly_available_assembly_details.txt which details the source, provider, WormBase assembly version used, its Bioproject ID, and the FTP address for easy download.
****

- Tables 1 and 2 in the main text should also be reformatted. Shading is not permitted by Gigascience. Also, removing vertical lines (both tables) and centering the numbers on table 1 would help to improve their look.

**********Response*******************
Done- many thanks for the feedback
****

- Please ensure that SRA and INSDC accessions are added, since they are currently referenced as "XXXXXXX"

**********Response*******************
Please see comment above to reviewer # 1
****

- Since InterProScan was ran, it would be interesting to look at the statistics in regards to the identification of InterPro domains. For example, compare the number of proteins with any annotated IPR domains, the total number of IPR domains identified, and the number of unique IPR domains identified. The previous publication also performed this comparison with other species using KEGG, so it may be interesting to repeat that similar analysis with the current annotation, although there are many updated ways to run KEGG so the re-analysis of the previous annotation may not match what was previously found.

**********Response*******************
Thank you for this suggestion. Extraction of these interproscan statistics did provide further encouraging results. We have included a Supporting data file called IPR.domain.analysis.txt containing the suggested Interproscan statistics and a paragraph has been added to the text to describe the results. We do not feel that the original Kegg analysis in the published paper generated meaningful biological insights and have therefore not replicated it here.
****

Powered by Editorial Manager® and ProduXion Manager® from Aries Systems Corporation

Reviewer #3:

Dear authors,

thank you for publishing the re-annotation of Heterorhabditis bacteriophora. It is both interesting for the particular research community dealing with Heterorhabditis bacteriophora, as well as for all research communities dealing with non-model organisms, in general. You demonstrate that the software applied for annotating a species can heavily impact conclusions drawn from a genome annotation project; and that it is worth re-annotating also non-model organisms with state of the art tools.

Below, you find my review, structured according to the Guide for GigaScience reviewers.

1. Is the rationale for collecting and analyzing the data well defined?

Yes.

2. Is it clear how data was collected and curated?

Yes, it is very clear.

3. Is it clear - and was a statement provided - on how data and analyses tools used in the study can be accessed?

For data, it is very clear.

The authors also make an effort to demonstrate tool availability (not their own, but software developed by others) by providing RRIDs. However, in some cases, the provided RRIDs are more confusing than helpful.

RRID:SCR_008419 is given for BLAST v2.6.0+ but the RRID leads to an URL that is not available (and in the past, when it was available, it corresponded to a particular BLAST interface for balsting against Aedes aegypti, an organsim that is not relevant to the manuscript under review). In this case, it would be more helpful to provide e.g. an URL to the download location of BLAST v2.6.0+; or create a new RRID.

**********Response*******************
Apologies for this error, thank you for noting it. A URL for downloading BLAST v2.6.0 has been provided and the incorrect RRID removed.
****

RRID:SCR_005622 is given for the RNA-Seq aligner STAR; the RRID leads to an URL for a user/password protected STAR related web application. I strongly assume the authors ran STAR locally, and thus, an URL to the offical STAR website would be more appropriate (https://github.com/alexdobin/STAR/releases), or the creation of a new RRID.

**********Response*******************
RRID corrected to the official website version

****
For Rstudio, accidentally, the RRID to STAR web application is provided. Please update to correct RRID or URL.

**********Response*******************
Corrected- thank you again for noting this error.
****

(No RRID or URL is provided for BRAKER. The URL is available in the referenced manuscript, though, and I believe that is sufficient. However, if journal policy is to always print RRIDs or URLs, you might want to add one of the download URLs. Also, BRAKER1 is the only tool where to do not list the version number (braker.pl --version).)

Powered by Editorial Manager® and ProduXion Manager® from Aries Systems Corporation

Version added.

4. Are accession numbers given or links provided for data that, as a standard, should be submitted to a community approved public repository?

In principle, yes, some accession numbers were still missing during the review process but will be updated by the authors prior publication.

5. Is the data and software available in the public domain under a Creative Commons license?

Scripts implemented particularly for this publication are avaiable at github, the license is GNU Public License V3. There are differences between licenses, I kindly ask the journal to check whether GPL fulfills the journal's requirements.

6. Are the data sound and well controlled?

Yes.

7. Is the interpretation (Analysis and Discussion) well balanced and supported by the data?

Yes.

8. Are the methods appropriate, well described, and include sufficient details and supporting information to allow others to evaluate and replicate the work?

In principle: yes. However, it might be useful to the community to provide not only references to the particular tool and version, but also the exact command lines that were used in this project. It would be really nice if you added the command lines to some supplementary document. For example, a reader who knows that BRAKER1 software, will assume that braker was called with the option --softmasking when the authors state that it was applied to a softmasked genome. A reader who is less familiar with the software will maybe not know this and might thus not be able to replicate the experiments, exactly.

**********Response*******************
Thank you for this suggestion, we agree and a Supplementary note has been added to this effect.
****

9. What are the strengths and weaknesses of the methods?

The authors used state of the art methods in a very suitable way.

10. Have the authors followed best-practices in reporting standards?

Yes.

11. Can the writing, organization, tables and figures be improved?

I am not a native speaker of English, myself, but I believe the language is good.

I hope that 1.747 as number of protein coding genes predicted by BRAKER1/soft-masked in Table 2 is a typo, please fix.

**********Response*******************
Corrected- thank you for spotting this
****

12. When revisions are requested.

Minor revisions:

Powered by Editorial Manager® and ProduXion Manager® from Aries Systems Corporation

	Please correct used software accessiblity references as recommended in point 3.- corrected as above<br><br>Please correct typo in Table 2 (point 11).- corrected as above.<br><br>Discretionary revisions:<br><br>Please consider my statement to point 8.- corrected as above<br><br>The journal should probably have a look at the license issue (point 5).<br><br>13. Are there any ethical or competing interests issues you would like to raise?<br><br>No.<br><br>I hope you find this review useful.<br><br>Kind regards,<br><br>Katharina Hoff
**Additional Information:**	
**Question**	**Response**
Are you submitting this manuscript to a special series or article collection?	No
**Experimental design and statistics**<br><br>Full details of the experimental design and statistical methods used should be given in the Methods section, as detailed in our Minimum Standards Reporting Checklist. Information essential to interpreting the data presented should be made available in the figure legends.<br><br>Have you included all the information requested in your manuscript?	Yes
**Resources**<br><br>A description of all resources used, including antibodies, cell lines, animals and software tools, with enough information to allow them to be uniquely identified, should be included in the Methods section. Authors are strongly encouraged to cite Research Resource Identifiers (RRIDs) for antibodies, model organisms and tools, where possible.<br><br>Have you included the information requested as detailed in our Minimum Standards Reporting Checklist?	Yes

**Availability of data and materials**<br><br>All datasets and code on which the conclusions of the paper rely must be either included in your submission or deposited in publicly available repositories (where available and ethically appropriate), referencing such data using a unique identifier in the references and in the “Availability of Data and Materials” section of your manuscript.<br><br>Have you have met the above requirement as detailed in our Minimum Standards Reporting Checklist?	Yes

Powered by Editorial Manager® and ProduXion Manager® from Aries Systems Corporation

Manuscript
Click here to download Manuscript ImprovingannotationofHbactED22-3.docx

Improving the annotation of the *Heterorhabditis bacteriophora* genome

Florence McLean[1], Duncan Berger[1], Dominik R. Laetsch[1], Hillel T. Schwartz[2] and Mark Blaxter[1]

1 Institute of Evolutionary Biology, University of Edinburgh, Edinburgh EH9 3JT, UK

2 Division of Biology and Biological Engineering, California Institute of Technology, Pasadena, California, USA

email addresses and ORCID

Florence McLean	f.e.mclean@sms.ed.ac.uk	0000-0001-8218-4904
Duncan Berger	duncan.berger@bath.edu	0000-0002-2017-6623
Dominik R. Laetsch	dominik.laetsch@gmail.com	0000-0001-7887-0186
Hillel Schwartz	hillels@caltech.edu	0000-0002-3448-8652
Mark Blaxter	mark.blaxter@ed.ac.uk	0000-0003-2861-949X

Abstract

Background: Genome assembly and annotation remains an exacting task. As the tools available for these tasks improve, it is useful to return to data produced with earlier instances to assess their credibility and correctness. The entomopathogenic nematode *Heterorhabditis bacteriophora* is widely used to control insect pests in horticulture. The genome sequence for this species was reported to encode an unusually high proportion of unique proteins and a paucity of secreted proteins compared to other related nematodes. **Findings:** We revisited the *H. bacteriophora* genome assembly and gene predictions to ask whether these unusual characteristics were biological or methodological in origin. We mapped an independent resequencing dataset to the genome and used the blobtools pipeline to identify potential contaminants. While present (0.2% of the genome span, 0.4% of predicted proteins), assembly contamination was not significant. **Conclusions:** Re-prediction of the gene set using BRAKER1 and published transcriptome data generated a predicted proteome that was very different from the published one. The new gene set had a much reduced complement of unique proteins, better completeness values that were in line with other related species' genomes, and an increased number of proteins predicted to be secreted. It is thus likely that methodological issues drove the apparent uniqueness of the initial *H. bacteriophora* genome annotation and that similar contamination and misannotation issues affect other published genome assemblies.

Background

The sequencing and annotation of a species' genome is often but the first step in exploiting these data for comprehensive biological understanding. As with all scientific endeavour, genome sequencing technologies and the bioinformatics toolkits available for assembly and annotation are being continually improved. It should come as no surprise therefore that first estimates of genome sequences and descriptions of the genes they contain can be improved. For example, the genome of the nematode *Caenorhabditis elegans* was the first animal genome to be sequenced [1]. The genome sequence and annotations have been updated many times since, as further exploration of this model organism revealed errors in original predictions, such that today, with release WS260 [2] [3], very few of the 19099 protein coding genes announced in the original publication [1] retain their original structure and sequence. The richness of the annotation of *C. elegans* is driven by the size of the research community that uses this model species. However for most species, where the community using the genome data is small or less-well funded, initial genome sequences and gene predictions are not usually updated.

Heterorhabditis bacteriophora is an entomopathogenic nematode which maintains a mutualistic association with the bacterium *Photorhabdus luminescens*. Unlike many other parasitic nematodes, it is amenable to *in vitro* culture [4] and is therefore of interest not only to evolutionary and molecular biologists investigating parasitic and symbiotic systems, but also to those concerned with the biological control of insect pests [5, 6]. *P. luminescens* colonises the anterior intestine of the free-living infective juvenile stage (IJ). IJs are attracted to insect prey by chemical signals [7, 8]. On contacting a host, the IJs invade the insect's haemocoel and actively regurgitate *P.*

luminescens into the haemolymph. The bacterial infection rapidly kills the insect, and *H. bacteriophora* grow and reproduce within the cadaver. After 2-3 cycles of replication, the nematode progeny develop into IJs, sequester *P. luminescens* and seek out new insect hosts.

Axenic *H. bacteriophora* IJs are unable to develop past the L1 stage [9] , and *H. bacteriophora* may depend on *P. luminescens* for secondary metabolite provision [10, 11]. Mutation of the global post-transcriptional regulator Hfq in *P. luminescens* reduced the bacterium's secondary metabolite production and led to failed nematode development, despite the bacterium maintaining virulence against host (*Galleria mellonella*) larvae [12]. Together these symbionts are efficient killers of pest (and other) insects, and understanding of the molecular mechanisms of host killing could lead to new insecticides.

H. bacteriophora was selected by the National Human Genome Research Initiative as a sequencing target [13]. Genomic DNA from axenic cultures of the inbred strain *H. bacteriophora* TTO1 was sequenced using Roche 454 technology and a high quality 77 Mb draft genome assembly produced [14]. This assembly was predicted (using JIGSAW [15]) to encode 21250 proteins. Almost half of these putative proteins had no significant similarity to entries in the GenBank non-redundant protein database, suggesting an explosion of novelty in this nematode. The predicted *H. bacteriophora* proteome had fewer orthologues of Kyoto Encyclopedia of Genes and Genomes loci in the majority of metabolic categories than nine other nematodes. *H. bacteriophora* was also predicted to have a relative paucity of secreted proteins compared to free-living nematodes, postulated to reflect a reliance on *P. luminescens* for secreted effectors [14]. The 5.7 Mb genome of *P. luminescens* has also been sequenced [16]. The *H. bacteriophora* proteome had fewer shared orthologues when clustered and

compared to other rhabditine (Clade V) nematodes (including *Caenorhabditis elegans* and the many animal parasites of the Strongylomorpha) [17].

In preliminary analyses we noted that while the genome sequence itself had high completeness scores when assessed with the Core Eukaryote Gene Mapping Approach (CEGMA) [18] (99.6% complete) and Benchmarking Universal Single-Copy Orthologs (BUSCO) [19] (80.9% complete and 5.6% fragmented hits for the BUSCO Eukaryota gene set), the predicted proteome scored poorly (47.8% complete and 34.7% fragmented by BUSCO; see Table 2). Another unusual feature of the *H. bacteriophora* gene set was the proportion of non-canonical splice sites (i.e. those with a 5' GC splice donor site, as opposed to the normal 5' GT). Most nematode (and other metazoan) genomes have low proportions of non-canonical introns (less than 1%) [20], but the published gene models had over 9% non-canonical introns. This is more than double the proportion predicted for *Globodera rostochiensis*, a plant parasitic nematode where the unusually high proportion of non-canonical introns was validated *via* manual curation [20].

If these unusual characteristics reflect a truly divergent proteome, the novel proteins in *H. bacteriophora* may be crucial in its particular symbiotic and parasitic relationships, and of great interest to development of improved strains for horticulture. However, it is also possible that contamination of the published assembly or annotation artefacts underpin these unusual features. We re-examined the *H. bacteriophora* genome and gene predictions, and used more recent tools to re-predict protein coding genes from the validated assembly. As the BRAKER1 predictions were demonstrably better than the original ones, we explored whether some of the unusual characteristics of the published protein set, in particular the level of novelty and the proportion of secreted proteins, were supported by the BRAKER1 protein set.

Findings

No evidence for substantial contamination of the *H. bacteriophora* genome assembly

We used BlobTools [21] to assess the published genome sequence [14] for potential contamination. The raw read data from the published assembly was not available on the trace archive or short read archive (SRA). We thus utilised new Illumina short-read re-sequencing data generated from strain G2a1223, an inbred derivative of *H. bacteriophora* strain "Gebre", isolated by Adler Dillman in Moldova. G2a1223 has about 1 single-nucleotide change per ~2000 nucleotides compared to the originally-sequenced TT01 strain. G2a1223 was grown in culture on the non-colonising bacterium *Photorhabdus temperata*. The majority of these data (96.3% of the reads) mapped as pairs to the assembly, suggesting completeness of the published assembly with respect to the new raw read data. In addition, 99.96% of the published assembly had at least 10-fold coverage from the new raw reads.

The assembly was explored using a taxon-annotated GC-coverage plot, with coverage taken from the new Illumina data and sequence similarity from the NCBI nucleotide (nt) database (Figure 1). *H. bacteriophora* was excluded from the database search used to annotate the scaffolds to exclude self hits from the published assembly. All large scaffolds clustered congruently with respect to read coverage and CG content. A few (57) scaffolds had best BLASTn matches to phyla other than Nematoda (Table 1). A small amount (5 kb) of likely remaining *P. luminescens* contamination was noted. We identified 100 kb of the genome of a strain of the common culture contaminant bacterium *Stenotrophomonas maltophilia* [22]. Contamination of the assembly with *S. maltophilia* was acknowledged [14] but removal of scaffolds before annotation was not

discussed. Two high-coverage scaffolds that derived from the *H. bacteriophora* mitochondrial genome were annotated as “undefined Eukaryota” because of taxonomic misclassification in the NCBI nt database. Many scaffolds with coverages close to that of the expected nuclear genome had best matches to two unexpected sources: the platyhelminths *Echinostoma caproni* and *Dicrocoelium dendriticum*, and several hymenopteran arthropods. Inspection of these matches showed that they were due to high sequence similarity to a family of *H. bacteriophora* mariner-like transposons [23] and thus these were classified as *bona fide* nematode nuclear sequences. A group of scaffolds contained what appears to be a *H. bacteriophora* nuclear repeat with highest similarity to histone H3.3 sequences from Diptera and Hymenoptera. The remaining scaffolds had low-scoring nucleotide matches to a variety of chordate, chytrid and arthropod sequences from deeply conserved genes (tubulin, kinases), but had coverages similar to other nuclear sequences.

Scaffolds with average coverage of less than 10-fold were removed from the assembly (35 scaffolds spanning 132949 bases, 0.2% of the total span; see Supporting Data [24]: *Low_coverage_scaffolds.txt*). This removed all scaffolds aligning to *S. maltophilia* and to *Photorhabdus* spp. (104 kb). The origins of the additional 28 kb were not investigated. In the published annotation [14], 76 genes were predicted from these scaffolds.

Improved gene predictions are biologically credible and have unexceptional novelty

New gene predictions were generated from a soft-masked version of the filtered assembly using the RNA-seq based annotation pipeline BRAKER1 v1.9 [25], generating 16070 protein predictions from 15747 protein coding genes (see

Supporting Data [24]: *BRAKER1.soft.masked.output.files.zip*). We compared the soft-masked predictions to those from the published analysis [14] (Figure 2, Table 2). The predicted proteins from the new BRAKER1/soft-masked gene set were, on average, longer (Figure 2A). While the average number of introns per gene was the same in the BRAKER1/soft-masked and published predictions, the BRAKER1/soft-masked gene set had more single-exon genes (Figure 2B). Hard masking of the genome and re-prediction resulted in fewer single exon genes, suggesting that many of these putative genes could be derived from repetitive sequence (Supporting Data [24]: *BRAKER1.hard.masked.output.files.zip* and *BRAKER1_annotation_comparisons.txt*), but only 316 of the single exon genes from the BRAKER1/soft-masked assembly had similarity to transposases or transposons. The BRAKER1/soft-masked annotations were taken forward for further analysis.

Four-fifths (83.3%) of the published protein-coding gene predictions [14] overlapped to some extent with the BRAKER1/soft-masked predictions at the genome level, with a mean of 67% of the nucleotides of each BRAKER1/soft-masked gene covered by a published gene (Figure 2C). Half (8061) of the 15747 BRAKER1/soft-masked gene predictions had an overlap proportion of ≥0.9 with the published predictions. At the level of protein sequence only 836 proteins were identical between the two predictions, and only 2099 genes had identical genome start and stop positions.

The BRAKER1/soft-masked and published gene sets were checked for completeness using BUSCO [19], based on the Eukaryota lineage gene set, and *Caenorhabditis* as the species parameter for orthologue finding. The BRAKER1/soft-masked gene set contained a substantially higher percentage of complete, and lower percentage of fragmented BUSCO genes than the published set (Table 2). Two *H. bacteriophora*

transcriptome datasets, publicly available Roche 454 data and Sanger expressed sequence tags, were mapped to the published and BRAKER1/soft-masked transcriptomes to assess gene set completeness. This suggested that the BRAKER1/soft-masked transcriptome predictions were more complete than the original (Table 2).

Nearly half (9893/20964; 47.2%) of the published proteins were reported to have no significant matches in the NCBI non-redundant protein database (nr) [14]. This surprising result could be due to a paucity of data from species closely related to *H. bacteriophora* in the NCBI nr database at the time of the search, or inclusion of poor protein predictions in the published set, or both. Targeted investigation of these 9893 orphan proteins here was not possible due to inconsistencies in gene naming in the publically available files. The published and BRAKER1/soft-masked proteomes were compared to the Uniref90 database [26], using DIAMOND v0.9.5 [27] with an expectation value cut-off of $1e^{-5}$. In the published proteome, 8962 proteins (42.7%) had no significant matches in Uniref90. Thus a relatively poorly populated database was not the main driver for the high number of orphan proteins reported in the published proteome. In the BRAKER1/soft-masked proteome, only 2889 proteins (18.3%) had no hits in the Uniref90 database (Table 2).

OrthoFinder v1.1.4 [28] was used to define orthologous groups in the proteomes of 23 rhabditine (Clade V) nematodes (Supporting Data [24]: *Orthofinder_analysis*) and just the published *H. bacteriophora* protein-coding gene predictions, or just the BRAKER/soft-masked proteome, or both. All proteins <30 amino-acids long were excluded from clustering (see Supporting: *Orthofinder_analysis*). We identified 5442 singletons (26.8% of the proteome) when the analysis included only the published *H. bacteriophora* protein set. An additional 248 proteins formed *H. bacteriophora*-specific

orthogroups. Orthology analysis including only the BRAKER/soft-masked protein set predicted 1112 *H. bacteriophora* singletons (7.1% of the proteome) with 167 proteins in *H. bacteriophora*-specific orthogroups (Figure 2D). In comparison, when the orthology analysis included the BRAKER1/soft-masked predictions there were 1858 *C. elegans* singletons (9.2% of the *C. elegans* proteome). Very few universal, single copy orthologues were defined in either analysis. Exploring "fuzzy-1-to-1" orthogroups (where true 1-to-1 orthology was found for greater than 75% of the 24 species - i.e. 18 or more species), the published protein predictions had more missing fuzzy-1-to-1 orthologues than did the BRAKER1/soft-masked predictions (Table 2). In the clustering that included both proteomes, 2019 clusters contained more proteins from the BRAKER1/soft-masked than the published proteome, whereas 2714 contained a larger number contributed from the published than the BRAKER1/soft-masked proteome (Supporting Data [24]: *kinfin.zip*).

The published *H. bacteriophora* gene set had additional peculiarities. The published set of gene models included 102274 introns, 9069 of which (8.9%) had non-canonical splice sites (i.e. 5' GC – AG 3'). Some of the genes in the published gene set had up to nine noncanonical introns (Figure 2E). In the BRAKER1/soft-masked gene set there were 109767 introns, 868 (0.8%) of which had non-canonical splice sites. This proportion is in keeping with that found in most other rhabditine nematodes. For example, the extensively manually annotated *C. elegans* has 2429 (0.6%) non-canonical (5' GC – AG 3') introns. In *C. elegans* non-canonical introns are frequently found only in alternately spliced, and shorter isoforms, and over 93-99% were in genes that had homologues in other species, depending on the species used in the protein orthology clustering. However, in the published *H. bacteriophora* gene set, 34-49% of the genes with GC – AG introns were in *H. bacteriphora*-unique proteins.

A supermatrix maximum likelihood phylogeny was generated from the fuzzy-1-1 orthologues in the clustering that included both *H. bacteriophora* proteomes (Figure 3; see Supporting Data [24]: *Phylogenetic_analyses*). The phylogeny, rooted with *Pristionchus* spp., shows the *H. bacteriophora* proteomes as sisters. However the BRAKER1/soft-masked proteome has a shorter branch length to *Heterorhabditis*' most recent common ancestor with other Clade V nematodes, suggesting that the published proteome includes uniquely divergent sequences.

The secretome of *H. bacteriophora* has been of particular interest as it may contain proteins involved in symbiotic interactions with *P. luminescens*, and proteins crucial to invasion and survival within the insect haemocoel. In the original publication, only 603 proteins (2.8% of the proteome) were predicted to be secreted [14]. This proportion is much lower than in free living nematodes such as *C. elegans* and it was postulated that *H. bacteriophora* relies on *P. luminescens* for secreted effectors [14]. The signal peptide detection method used in the original analyses was not described [14]. We used SignalP version 4.1 within Interproscan to annotate proteins in both the BRAKER1 and published *H. bacteriophora* proteomes. Proteins having a predicted signal peptide but no transmembrane domain were classified as secreted. We identified 1023 (6.5%) putative secreted proteins in the BRAKER1/soft-masked proteome and 1067 (5.1%) in the published proteome. By the same method other rhabditine (Clade V) nematodes that do not have known symbiotic associations with bacteria, such as *Teladorsagia circumcincta*, had comparable secretome sizes to *H. bacteriophora* (Supporting Data [24]: *Secretome_analysis.txt*). This suggests that *H. bacteriophora* does not have a reduced secretome compared to other, related nematodes that do not have symbiont partners.

Interproscan was also used to annotate the BRAKER1 and published proteomes by identifying matches against the databases TIGRFAM v15.0, ProDom v2006.1, SMART-7.1, PrositePatterns v20.119, PRINTS v42.0, SuperFamily v1.75, Pfam v29.0, and PrositeProfiles v20.119. The BRAKER1 proteome had a greater number of proteins annotated with at least one domain compared to the published proteome, and a greater number of total domains identified (Supporting Data [24]: *IPR.domain.analysis.txt*).

Discussion

Assembly of, and genefinding in, new genomes is a challenging task, and especially so in larger genomes and those phylogenetically distant from any previously analysed exemplar. When applied *de novo* to datasets from extremely well-assembled and well-annotated model species, even the best methods fail to recover fully contiguous assemblies and yield predicted gene sets that have poor correspondence with the known truth [29]. A major issue with primary assemblies and gene sets arises when exceptional findings are taken at face value, and used to assert exceptional biology in a target species [30]. Where these exceptions are in fact the result of methodological failings, the scientific record, including the public databases, becomes contaminated. At best, erroneous assertions can be quickly checked and corrected, but at worst they can mislead and inhibit subsequent work.

A second concern arises from the recognition that while no method can currently produce perfect assemblies and perfect gene sets from raw data, analyses using the same toolsets will resemble each other and reflect the successes and failings of the particulars of the algorithms employed. However, when comparing genome assemblies and gene sets produced by different pipelines, it may be that the disparity

in output generated by different pipelines dominates any signal from biology. Genomes assembled and annotated with the same tools will look more similar, and in a pool of assemblies and protein sets the one species that used a variant process will be flagged as exceptional. Again, the model organisms show the way: as new data and new scrutiny is added to the genome, better and better analyses are available. With additional analysis, and additional independent data, genome and gene predictions can be improved markedly for any species [31].

Here we examined the “outlier” whole-genome protein predictions from the entomopathogenic nematode *H. bacteriophora* [14]. The original publication noted that the number of novel proteins (those restricted to *H. bacteriophora*) was particularly large, while the number of secreted proteins was rather small, and suggested that these genome features might be a result of evolution to the species’ novel lifestyle (which includes an essential symbiosis with the bacterium *P. luminescens*). Overall we found that while the published genome sequence had a small amount of bacterial contamination, and a small number of “nematode” genes were predicted from these contaminants, the assembly itself was of high quality. Our re-prediction of the gene set of *H. bacteriophora* however suggested that the excess of unique genes, the lack of secreted proteins and several other surprising features of the original gene set were likely to be artefacts of the gene prediction pipeline chosen. While our gene set was by no means perfect (for example we identified an excess of single exon genes that derive from likely repetitive sequence) it had better biological completeness and credibility.

We used the RNA-seq based annotation pipeline BRAKER1 [25], not available to the authors of the original genome publication, who used JIGSAW [15] (see Supplementary File 1). While JIGSAW achieved high sensitivity and specificity at the

level of nucleotide, exon and gene predictions in the nematode genome annotation assessment project, nGASP [29], direct comparison of the sensitivity and specificity of JIGSAW and BRAKER1 has not been published to the best of our knowledge. BRAKER1 has been shown to give superior prediction results over *ab initio* GeneMark-ES, or *ab initio* AUGUSTUS alone [25]. In particular, BRAKER1 is able to better use transcriptome data for gene finding. While we supplied only a partial Roche 454 transcriptome to BRAKER1, the resulting gene set has much improved numerical and biological scores. In particular we note that the biological completeness of the predicted gene set now matches that of the genome sequence from which it was derived (Table 2).

The published gene set had an unusually high proportion (8.9%) of non-canonical (5' GC – AG 3') introns. While most genomes have a low proportion of non-canonical introns (usually approximately 0.5% of all introns), some species have markedly higher proportions [20]. The high proportion found initially in *H. bacteriophora* could perhaps have been taken as a warning that the prediction set was of concern. We note that gene predictors can be set to disallow any predictions that require non-canonical splicing, and many published genomes have zero non-canonical introns. These gene prediction sets are likely to categorically miss true non-canonically spliced genes.

The new BRAKER1 gene prediction set had many fewer species-unique genes (7.1%) than did the original (42.7%) when compared to 23 other related nematodes. We regard this reduction in novelty as indicative of a better prediction, as, for example, *C. elegans*, the best-annotated nematode genome, had only 9.2% of species unique genes in our analysis. Having a large proportion of orphan proteins is not unique to the published *H. bacteriophora* predictions. Nearly half (47%) of the gene predictions in *Pristionchus pacificus* were reported to have no homologues in fifteen other

nematode species [32]. Evaluation of proteomic and transcriptomic evidence, as well as patterns of synonymous and non-synonymous substitution, suggested that as many as 42-81% of these genes were in fact expressed [33]. Therefore the high proportion of orphan genes in *H. bacteriophora* is not *prima facie* evidence of poor gene predictions. Expanded transcriptomic and comparative data are needed to build on the work we have presented in affirming the true *H. bacteriophora* gene set.

Biological pest control agents may become increasingly important for ensuring crop protection in the future [34]. A number of factors currently limit the commercial applicability of *H. bacteriophora*, including their short shelf life, susceptibility to environmental stress and limited insect tropism [13, 35]. Accurate genome annotation will assist in the analysis of *H. bacteriophora*, facilitating the exploration of genes involved in its parasitic and symbiotic interactions, and supporting genetic manipulation to enhance its utility as a biological control agent.

Methods

Methods Supplementary Note

A detailed description of the command lines used in the generation of the BRAKER1 gene predictions and the associated analysis can be found in Supplementary File 2 which accompanies this manuscript.

Contaminant screening and Removal of Low Coverage Scaffolds

The assembly scaffolds were aligned to the NCBI nt database, release 204, using Nucleotide-Nucleotide BLAST v2.6.0+ (available at:[36]) in megablast mode, with an e-value cut off of $1e^{-25}$ and a culling limit of 2 [37]. *H. bacteriophora* hits were excluded from the search using a list of all *H. bacteriophora* associated gene identifiers downloaded from NCBI GenBank nucleotide database, release 219. Raw, paired-end Illumina reads from the re-sequencing project were mapped against the assembly, as paired, using Burrows-Wheeler Aligner (BWA) v0.7.15 (available at:[38]) in mem mode with default options [39]. The output was converted to a BAM file using Samtools v1.3.1 (SAMTOOLS, RRID:SCR_002105) [40] and overall mapping statistics generated in flagstat mode.

Blobtools v0.9.19 [21] was used to create taxon annotated GC-coverage plots for the published assembly, using the Nucleotide-Nucleotide BLAST and raw read mapping results. Scaffolds that did not have Nematoda as a top BLAST hit at the phylum level were identified, and the species-level top BLAST hit, length of scaffold, and scaffold mean base coverage were extracted from the Blobology output. Scaffolds with a mean base coverage of <10x were identified from the output of the Blobology pipeline and removed from the assembly. A list of excluded scaffolds is available in Supporting Data [24]*: Low_coverage_scaffolds.txt*.

Generation of BRAKER1 Gene Predictions

Before annotation the published assembly was soft-masked for known Nematoda repeats from the RepeatMasker Library v4.0.6 using RepeatMasker v4.0.6 (RepeatMasker, RRID:SCR_012954) [41] with default options. The two publicly available Roche 454 RNA-seq data files were adaptor and quality-trimmed using BBDuk v36.92 (unpublished toolkit from Joint Genome Institute, n.d.). Reads below an average quality of 10 or shorter than 25 nucleotides were discarded. Regions with average quality below 20 were trimmed. The cleaned reads were mapped to the soft-masked assembly using STAR v2.5 (STAR, RRID:SCR_015899) with default options [42, 43]. The soft-masked assembly was annotated with BRAKER1 v1.9 [25] with guidance from the mapping output from STAR. An identical annotation method was applied to a hard-masked version of the assembly. The assembly was hard-masked for known Nematoda repeats from the RepeatMasker Library v4.0.6 using RepeatMasker v4.0.6 with default options. The published and BRAKER1 proteomes were compared using DIAMOND v0.9.5 [27] in BLASTP mode to the Uniref90 database (release 03/2017) [26] with an expectation value cut-off of $1e^{-5}$ and no limit on the number of target sequences. Hits to *H. bacteriophora* proteins were removed using its TaxonID.

Gene Prediction Statistics

Gene-level statistical summaries were calculated including only the longest isoforms of the BRAKER1 gene predictions. The longest isoform for each gene in the BRAKER1 *H. bacteriophora* annotation was identified from the general feature format (GFF) file, and then selected from the protein FASTA files. The GFF file for the published gene predictions did not contain any isoforms and was analysed in its entirety. f Introns were

inferred for the published GFF file using GenomeTools v1.5.9 in -addintrons mode [44]. Intron frequencies were then calculated for the published and BRAKER1 annotations from their respective GFF files. Exon frequencies were calculated for the published annotations directly from the GFF file. For the BRAKER1 annotations, exon frequency per gene was assumed to be equivalent to coding DNA sequence (CDS) frequency and inferred from the GFF file, as exon features were not included in the GFF file. Intron frequency histograms and bar plots were generated in Rstudio v1.0.136 (RStudio, RRID:SCR_000432) with R v3.3.2 (R Project for Statistical Computing, RRID:SCR_001905) and in some instances the package ggplot2 v2.2.1. As intron frequency lists did not contain single exon genes (those with no introns), these were added manually to the intron frequency lists in Microsoft Excel before importing the data into Rstudio.

The proportion of introns with GC – AG splice junctions was assessed for the gene models of *C. elegans* (WS258), and the published and BRAKER1/soft-masked gene models of *H. bacteriophora*. Intronic features were added to general feature format version 3 (GFF3) files using GenomeTools v1.5.9 [44] ('gt gff3 -sort -tidy -retainids –fixregionboundaries -addintrons') and splice sites were extracted using the script extractRegionFromCoordinates.py [20]. Results were visualised using the script plot_GCAG_counts.R (available at: [45]).

Gene features, extracted from the GFF files, were assessed for overlap using bedtools v2.26 (BEDTools, RRID:SCR_006646) in intersect mode [46]. Only genes on the same strand were considered to be overlapping. To calculate the number of identical proteins shared between the published and BRAKER1 proteomes non-redundant protein fasta files were generated using cd-hit v4.6.1 (CD-HIT, RRID:SCR_007105)

[47] for the BRAKER1 and published predictions. The files were concatenated, sorted and unique sequences counted using unix command line tools.

BUSCO v2.0.1 (BUSCO, RRID:SCR_015008) [19], with Eukaryota as the lineage dataset, and *Caenorhabditis* as the species parameter for orthologue finding was applied to both proteomes and the published assembly to calculate BUSCO scores. CEGMA (CEGMA, RRID:SCR_015055) [18] was run on the published genome sequence. BWA was used with default settings to map the RNA-seq datasets (the Sanger ESTs in assembled form) to the CDS transcripts from the published and BRAKER1 annotations and the summary statistics obtained with Samtools v1.3.1 in flagstat mode.

Protein orthology analyses

OrthoFinder v1.1.4 [28] with default settings was used to identify orthologous groups in the proteomes of 23 Clade V nematodes with the addition of either the BRAKER1/soft-masked and published *H. bacteriophora* proteomes separately or simultaneously. The proteomes for the 23 Clade V nematodes were downloaded from WBPS8 (available at:[48]) or GenomeHubs.org (available at: [49]), and detailed source information is available in Supporting Data [24]*: Secretome.analysis.txt*. All proteomes were filtered to contain only the longest isoform of each gene, and for all proteomes (except the BRAKER1/soft-masked *H. bacteriophora* protein set), proteins less than 30 amino-acids in length were excluded before clustering. For the *H. bacteriophora* BRAKER1/soft-masked protein set, proteins less than 30 amino-acids (SF5.2) were removed manually from the orthofinder clustering statistics after clustering. None of these proteins seeded new clusters and are therefore will not have influenced the clustering results. Kinfin v0.9 [50], was used with default settings to identify true and

fuzzy 1-to-1 orthologues, and their associated species specific statistics. Fuzzy 1-to-1 orthologues are true 1-to-1 orthologues for greater than 75% of the species clustered. For the clustering analysis presented in Supporting Data [24]: *Orthofinder_analysis*, the BRAKER1/soft-masked and published proteomes were clustered simultaneously to the 23 other Clade V nematode proteomes, and singletons, and species-specific clusters were excluded.

Interproscan and search for transposons

Interproscan v5.19-58.0 (RRID:SCR_005829) [51] was used in protein mode to identify matches in the BRAKER1 and published proteomes in the following databases: TIGRFAM v15.0, ProDom v2006.1, SMART-7.1, SignalP-EUK v4.1, PrositePatterns v20.119, PRINTS v42.0, SuperFamily v1.75, Pfam v29.0, and PrositeProfiles v20.119. For secretome analysis of the 23 Clade V nematodes Interproscan v5.19-58.0 was run against the SignalP-EUK v4.1 database alone. InterProScan was run with the option for all match calculations to be run locally and with gene ontology annotation activated. The number of single exon genes with similarity to transposons or transposases in the BRAKER1/soft-masked predictions was calculated by searching the full InterProScan results for the strings ‘Transposon’, ‘transposon’, ‘Transposase’, or ‘transposase’ and the number of single exon gene InterProScan results containing these terms counted. InterProScan results from searching the SignalP-EUK-4.1 database were queried to identify putative secreted proteins. Those with a predicted signal peptide but no transmembrane region were considered to be secreted.

Phylogenetic Analyses

Both *H. bacteriophora* proteomes were clustered simultaneously with the 23 Clade V nematode proteomes into orthologous groups using Orthofinder v1.0 [28]. The fuzzy 1-to-1 orthologues were extracted and processed using GNU parallel [52]. They were aligned using MAFFT v7.267 (RRID:SCR_011811) [53], and the alignments trimmed with NOISY v1.5.12 [54]. A maximum likelihood gene tree was generated for each orthologue using RaXML v8.1.20 (RRID:SCR_006086) with a PROTGAMMAGTR amino-acid substitution model [55]. Rapid Bootstrap analysis and search for the best -scoring ML tree within one program run with 100 rapid bootstrap replicates was used. The trees were pruned using PhyloTreePruner v1.0 [56] to remove paralogues, with 0.5 as the bootstrap cutoff and a minimum of 20 species in the orthogroup after pruning for inclusion in the supermatrix. Where species had more than one putative orthologue in an orthogroup the longest was selected. The remaining 897 orthogroups were re-aligned using MAFFT v7.267, trimmed with NOISY v1.5.12 and concatenated into a supermatrix using FASconCAT v.1.0 [57]. A supermatrix maximum-likelihood tree was generated using RAxML with the rapid hill climbing algorithm (default), with a PROTGAMMAGTR amino-acid substitution model and 100 bootstrap replicates . *Pristionchus* spp. were designated as the outgroup. The tree was visualised in Dendroscope v3.5.9 [58].

Input data and data availability

The *H. bacteriophora* genome and annotations [14] were downloaded from Wormbase *Parasite* *(WBPS8)* (see Supporting Data [24]: *Publicly_available_assembly_details.txt*). The ESTs [59, 60] were obtained from NCBI dbEST [61] (accessions listed in Supporting Data [24]: *EST.acc.txt*), and the

assembled versions used in the analysis are available in Supporting Data [24]: *EST.assembled.fas*. Roche 454 transcriptome data [14] were obtained from the Short Read Archive (Accession numbers: SRX001441 and SRX001440). *H. bacteriophora* strain Gebre, a gift from Adler Dillman, was inbred by selfing single hermaphrodites for five generations to generate the strain G2a1223. New Illumina HiSeq2000, paired end, 75 base data were generated from *H. bacteriophora* G2a1223 genomic DNA by the Millard and Muriel Jacobs Genetics and Genomics Laboratory at Caltech (Short Read Archive accession number: SRP135845).

The revised gene annotations for *H. bacteriophora* have been submitted to Zenodo [62]. The supporting data for this manuscript is additionally available via the GigaScience repository, GigaDB [24].

Competing interests

The authors declare that they have no competing interests

Funding

This project was supported by FMs Wellcome Trust-funded graduate programme [204052/Z/16/Z].

Author’s contributions

Conceptualization, MB; Methodology, FM, DB, and MB; Formal analysis, FM, DRL and MB; Supervision, MB; Writing- original draft, FM and DRL; Writing- review and editing, FM, MB, DRL, DB, HTS; Resources, HTS.

Acknowledgements

Sujai Kumar, Lewis Stevens, Carlos Caurcel and Elisabeth Sjokvist offered expert technical support and advice. Igor Antoshechkin of the Millard and Muriel Jacobs

Genetics and Genomics Laboratory at Caltech assisted with Illumina sequencing. Adler Dillman provided the parental strain for the inbred *H. bacteriophora* strain G2a1223.

References

1. C. elegans Sequencing Consortium. Genome sequence of the nematode C. elegans: a platform for investigating biology. Science (80-). 1998;282:2012–8.

2. WormBase, version WS260. http://www.wormbase.org/.

3. Howe KL, Bolt BJ, Cain S, Chan J, Chen WJ, Davis P, et al. WormBase 2016: expanding to enable helminth genomic research. Nucleic Acids Res. 2016;44:D774-80. doi:10.1093/nar/gkv1217.

4. Gil GH, Choo HY, Gaugler R. Enhancement of entomopathogenic nematode production in in-vitro liquid culture of Heterorhabditis bacteriophoraby fed-batch culture with glucose supplementation. Appl Microbiol Biotechnol. 2002;58:751–5. doi:10.1007/s00253-002-0956-1.

5. Memari Z, Karimi J, Kamali S, Goldansaz SH, Hosseini M. Are Entomopathogenic Nematodes Effective Biological Control Agents Against the Carob Moth,Ectomyelois ceratoniae? J Nematol. 2016;48:261–7.

6. Rezaei N, Karimi J, Hosseini M, Goldani M, Campos-Herrera R. Pathogenicity of Two Species of Entomopathogenic Nematodes Against the Greenhouse Whitefly, Trialeurodes vaporariorum (Hemiptera: Aleyrodidae), in Laboratory and Greenhouse

Experiments. J Nematol. 2015;47:60–6.

7. Dillman AR, Guillermin ML, Lee JH, Kim B, Sternberg PW, Hallem EA. Olfaction shapes host-parasite interactions in parasitic nematodes. Proc Natl Acad Sci USA. 2012;109:E2324-33. doi:10.1073/pnas.1211436109.

8. Anbesse S, Ehlers RU. Attraction of Heterorhabditis sp. toward synthetic (E)-beta-cariophyllene, a plant SOS signal emitted by maize on feeding by larvae of Diabrotica virgifera virgifera. Commun Agric Appl Biol Sci. 2010;75:455–8.

9. Han R, Ehlers RU. Pathogenicity, development, and reproduction of Heterorhabditis bacteriophora and Steinernema carpocapsae under axenic in vivo conditions. J Invertebr Pathol. 2000;75:55–8. doi:10.1006/jipa.1999.4900.

10. Ciche TA, Bintrim SB, Horswill AR, Ensign JC. A Phosphopantetheinyl transferase homolog is essential for Photorhabdus luminescens to support growth and reproduction of the entomopathogenic nematode Heterorhabditis bacteriophora. J Bacteriol. 2001;183:3117–26. doi:10.1128/JB.183.10.3117-3126.2001.

11. Bennett HPJ, Clarke DJ. The pbgPE operon in Photorhabdus luminescens is required for pathogenicity and symbiosis. J Bacteriol. 2005;187:77–84. doi:10.1128/JB.187.1.77-84.2005.

12. Tobias NJ, Heinrich AK, Eresmann H, Wright PR, Neubacher N, Backofen R, et al. Photorhabdus-nematode symbiosis is dependent on hfq-mediated regulation of secondary metabolites. Environ Microbiol. 2017;19:119–29. doi:10.1111/1462-2920.13502.

13. Ciche T. The biology and genome of Heterorhabditis bacteriophora. WormBook. 2007;:1–9. doi:10.1895/wormbook.1.135.1.

14. Bai X, Adams BJ, Ciche TA, Clifton S, Gaugler R, Kim K, et al. A lover and a fighter: the genome sequence of an entomopathogenic nematode Heterorhabditis bacteriophora. PLoS One. 2013;8:e69618. doi:10.1371/journal.pone.0069618.

15. Allen JE, Salzberg SL. JIGSAW: integration of multiple sources of evidence for gene prediction. Bioinformatics. 2005;21:3596–603. doi:10.1093/bioinformatics/bti609.

16. Duchaud E, Rusniok C, Frangeul L, Buchrieser C, Givaudan A, Taourit S, et al. The genome sequence of the entomopathogenic bacterium Photorhabdus luminescens. Nat Biotechnol. 2003;21:1307–13. doi:10.1038/nbt886.

17. Blaxter M, Koutsovoulos G. The evolution of parasitism in Nematoda. Parasitology. 2015;142 Suppl 1:S26-39. doi:10.1017/S0031182014000791.

18. Parra G, Bradnam K, Korf I. CEGMA: a pipeline to accurately annotate core genes in eukaryotic genomes. Bioinformatics. 2007;23:1061–7. doi:10.1093/bioinformatics/btm071.

19. Simão FA, Waterhouse RM, Ioannidis P, Kriventseva EV, Zdobnov EM. BUSCO: assessing genome assembly and annotation completeness with single-copy orthologs. Bioinformatics. 2015;31:3210–2. doi:10.1093/bioinformatics/btv351.

20. Eves-van den Akker S, Laetsch DR, Thorpe P, Lilley CJ, Danchin EGJ, Da Rocha M, et al. The genome of the yellow potato cyst nematode, Globodera rostochiensis, reveals insights into the basis of parasitism and virulence. Genome Biol. 2016;17:124. doi:10.1186/s13059-016-0985-1.

21. Laetsch DR, Blaxter ML. BlobTools: Interrogation of genome assemblies [version 1; referees: 2 approved with reservations]. F1000Res. 2017;6:1287.

doi:10.12688/f1000research.12232.1.

22. Fierst JL, Murdock DA, Thanthiriwatte C, Willis JH, Phillips PC. Metagenome-Assembled Draft Genome Sequence of a Novel MicrobialStenotrophomonas maltophiliaStrain Isolated fromCaenorhabditisremaneiTissue. Genome Announc. 2017;5. doi:10.1128/genomeA.01646-16.

23. Grenier E, Abadon M, Brunet F, Capy P, Abad P. A mariner-like transposable element in the insect parasite nematode Heterorhabditis bacteriophora. J Mol Evol. 1999;48:328–36.

24. McLean F, Berger D, Laetsch DR, Schwartz HT, Blaxter M. Supporting data for “Improving the annotation of the Heterorhabditis bacteriophora genome” GigaScience Database 2018. http://dx.doi.org/10.5524/100404 .

25. Hoff KJ, Lange S, Lomsadze A, Borodovsky M, Stanke M. BRAKER1: Unsupervised RNA-Seq-Based Genome Annotation with GeneMark-ET and AUGUSTUS. Bioinformatics. 2016;32:767–9. doi:10.1093/bioinformatics/btv661.

26. Boutet E, Lieberherr D, Tognolli M, Schneider M, Bansal P, Bridge AJ, et al. UniProtKB/Swiss-Prot, the Manually Annotated Section of the UniProt KnowledgeBase: How to Use the Entry View. Methods Mol Biol. 2016;1374:23–54. doi:10.1007/978-1-4939-3167-5_2.

27. Buchfink B, Xie C, Huson DH. Fast and sensitive protein alignment using DIAMOND. Nat Methods. 2015;12:59–60. doi:10.1038/nmeth.3176.

28. Emms DM, Kelly S. OrthoFinder: solving fundamental biases in whole genome comparisons dramatically improves orthogroup inference accuracy. Genome Biol. 2015;16:157. doi:10.1186/s13059-015-0721-2.

29. Coghlan A, Fiedler TJ, McKay SJ, Flicek P, Harris TW, Blasiar D, et al. nGASP--the nematode genome annotation assessment project. BMC Bioinformatics. 2008;9:549. doi:10.1186/1471-2105-9-549.

30. Koutsovoulos G, Kumar S, Laetsch DR, Stevens L, Daub J, Conlon C, et al. No evidence for extensive horizontal gene transfer in the genome of the tardigrade Hypsibius dujardini. Proc Natl Acad Sci USA. 2016;113:5053–8. doi:10.1073/pnas.1600338113.

31. Yoshida Y, Koutsovoulos G, Laetsch DR, Stevens L, Kumar S, Horikawa DD, et al. Comparative genomics of the tardigrades Hypsibius dujardini and Ramazzottius varieornatus. PLoS Biol. 2017;15:e2002266. doi:10.1371/journal.pbio.2002266.

32. Baskaran P, Rödelsperger C, Prabh N, Serobyan V, Markov GV, Hirsekorn A, et al. Ancient gene duplications have shaped developmental stage-specific expression in Pristionchus pacificus. BMC Evol Biol. 2015;15:185. doi:10.1186/s12862-015-0466-2.

33. Prabh N, Rödelsperger C. Are orphan genes protein-coding, prediction artifacts, or non-coding RNAs? BMC Bioinformatics. 2016;17:226. doi:10.1186/s12859-016-1102-x.

34. Kergunteuil A, Bakhtiari M, Formenti L, Xiao Z, Defossez E, Rasmann S. Biological Control beneath the Feet: A Review of Crop Protection against Insect Root Herbivores. Insects. 2016;7. doi:10.3390/insects7040070.

35. Ali F, Wharton DA. Cold tolerance abilities of two entomopathogenic nematodes, Steinernema feltiae and Heterorhabditis bacteriophora. Cryobiology. 2013;66:24–9. doi:10.1016/j.cryobiol.2012.10.004.

36. NCBI blast executables. ftp://ftp.ncbi.nlm.nih.gov/blast/executables/blast+/2.6.0/.

37. Boratyn GM, Camacho C, Cooper PS, Coulouris G, Fong A, Ma N, et al. BLAST: a more efficient report with usability improvements. Nucleic Acids Res. 2013;41 Web Server issue:W29-33. doi:10.1093/nar/gkt282.

38. Sourceforge.net. https://sourceforge.net/projects/bio-bwa/files/. Accessed 1 Apr 2017.

39. Li H, Durbin R. Fast and accurate short read alignment with Burrows-Wheeler transform. Bioinformatics. 2009;25:1754–60. doi:10.1093/bioinformatics/btp324.

40. Li H, Handsaker B, Wysoker A, Fennell T, Ruan J, Homer N, et al. The Sequence Alignment/Map format and SAMtools. Bioinformatics. 2009;25:2078–9. doi:10.1093/bioinformatics/btp352.

41. Tarailo-Graovac M, Chen N. Using RepeatMasker to identify repetitive elements in genomic sequences. Curr Protoc Bioinformatics. 2009;Chapter 4:Unit 4.10. doi:10.1002/0471250953.bi0410s25.

42. Dobin A, Davis CA, Schlesinger F, Drenkow J, Zaleski C, Jha S, et al. STAR: ultrafast universal RNA-seq aligner. Bioinformatics. 2013;29:15–21. doi:10.1093/bioinformatics/bts635.

43. Dobin A, Gingeras TR. Optimizing RNA-Seq Mapping with STAR. Methods Mol Biol. 2016;1415:245–62. doi:10.1007/978-1-4939-3572-7_13.

44. Gremme G, Steinbiss S, Kurtz S. GenomeTools: a comprehensive software library for efficient processing of structured genome annotations. IEEE/ACM Trans Comput Biol Bioinform. 2013;10:645–56. doi:10.1109/TCBB.2013.68.

45. Laetsch D. plot_GCAG_counts.R . https://github.com/DRL/mclean2017/tree/master/analysis/splice_sites.

46. Quinlan AR, Hall IM. BEDTools: a flexible suite of utilities for comparing genomic features. Bioinformatics. 2010;26:841–2. doi:10.1093/bioinformatics/btq033.

47. Huang Y, Niu B, Gao Y, Fu L, Li W. CD-HIT Suite: a web server for clustering and comparing biological sequences. Bioinformatics. 2010;26:680–2. doi:10.1093/bioinformatics/btq003.

48. WormBase ParasiteSite version 8. ftp://ftp.ebi.ac.uk/pub/databases/wormbase/parasite/releases/WBPS8/. Accessed 2 April 2017.

49. GenomeHubs.org. http://ensembl.caenorhabditis.org/index.html. Accessed 2 Apr 2017.

50. Laetsch DR, Blaxter ML. KinFin: Software for Taxon-Aware Analysis of Clustered Protein Sequences. G3 (Bethesda). 2017;7:3349–57. doi:10.1534/g3.117.300233.

51. Finn RD, Attwood TK, Babbitt PC, Bateman A, Bork P, Bridge AJ, et al. InterPro in 2017-beyond protein family and domain annotations. Nucleic Acids Res. 2017;45:D190–9. doi:10.1093/nar/gkw1107.

52. Tange O. GNU Parallel - The Command-Line Power Tool. login: The USENIX Magazine. 2011;36:42–7.

53. Katoh K, Standley DM. MAFFT multiple sequence alignment software version 7: improvements in performance and usability. Mol Biol Evol. 2013;30:772–80. doi:10.1093/molbev/mst010.

54. Dress AWM, Flamm C, Fritzsch G, Grünewald S, Kruspe M, Prohaska SJ, et al. Noisy: identification of problematic columns in multiple sequence alignments. Algorithms Mol Biol. 2008;3:7. doi:10.1186/1748-7188-3-7.

55. Stamatakis A. RAxML-VI-HPC: maximum likelihood-based phylogenetic analyses with thousands of taxa and mixed models. Bioinformatics. 2006;22:2688–90. doi:10.1093/bioinformatics/btl446.

56. Kocot KM, Citarella MR, Moroz LL, Halanych KM. PhyloTreePruner: A Phylogenetic Tree-Based Approach for Selection of Orthologous Sequences for Phylogenomics. Evol Bioinform Online. 2013;9:429–35. doi:10.4137/EBO.S12813.

57. Kück P, Meusemann K. FASconCAT: Convenient handling of data matrices. Mol Phylogenet Evol. 2010;56:1115–8. doi:10.1016/j.ympev.2010.04.024.

58. Huson DH, Scornavacca C. Dendroscope 3: an interactive tool for rooted phylogenetic trees and networks. Syst Biol. 2012;61:1061–7. doi:10.1093/sysbio/sys062.

59. Sandhu SK, Jagdale GB, Hogenhout SA, Grewal PS. Comparative analysis of the expressed genome of the infective juvenile entomopathogenic nematode, Heterorhabditis bacteriophora. Mol Biochem Parasitol. 2006;145:239–44. doi:10.1016/j.molbiopara.2006.01.002.

60. Hao Y-J, Montiel R, Lucena MA, Costa M, Simoes N. Genetic diversity and comparative analysis of gene expression between Heterorhabditis bacteriophora Az29 and Az36 isolates: uncovering candidate genes involved in insect pathogenicity. Exp Parasitol. 2012;130:116–25. doi:10.1016/j.exppara.2011.12.001.

61. Boguski MS, Lowe TM, Tolstoshev CM. dbEST--database for “expressed

sequence tags". Nat Genet. 1993;4:332–3. doi:10.1038/ng0893-332.

62. McLean F, Berger D, Laetsch DR, Schwartz HT,Blaxter M. Revised gene annotations for the entemopathogenic nematode,Heterorhabditis bacteriophora. Zenodo data repository. 10.5281/zenodo.1169646 .

Figures and Legends

Figure 1. Taxon-annotated GC-coverage plot of the *H. bacteriophora* assembly.

Bottom left panel: Each scaffold or contig is represented by a single filled circle. Each scaffold is placed in the main panel based on its GC proportion (X axis) and coverage by reads from the Illumina re-sequencing project (Y axis). The fill colour of the circle indicates the taxon of the top BLASTn hit in the NCBI nt database for that scaffold. The colours are annotated in the top right hand key, which indicates taxon assignment and (in brackets) the number of contigs and scaffolds so assigned, their total span, and their N50 length. The circles are scaled to scaffold length, as indicated in the key at the base of the main panel.

Right panel: Nucleotide span in kb at each coverage level.

Top panel: Nucleotide span in kb at each GC proportion.

Figure 2. Comparisons of BRAKER1/soft-masked and original gene predictions from *H. bacteriophora*

(A, B) Frequency histograms of intron count (A) and protein length (B) in BRAKER1/soft-masked (blue) and published (yellow) protein coding gene predictions.

Outlying proteins longer than >2500 amino-acids (n=40) or genes containing >60 introns (n=20) are not shown.

(C) Frequency histogram of the proportion of each BRAKER1 gene prediction overlapped by a published gene prediction at the nucleotide level.

(D) Comparison of singleton, proteome-specific, and shared proteins in the published and BRAKER1/soft-masked protein sets.

(E) Counts of non-canonical GC/AG introns in gene predictions from the published and BRAKER1 *H. bacteriophora* gene sets, and the model nematode *Caenorhabditis elegans* (WS258). Counts are of genes containing at least one non-canonical GC/AG intron with the specified number of non-canonical introns.

Figure 3. Maximum likelihood phylogeny of selected rhabditine (Clade V) nematodes.

A supermatrix of aligned amino acid sequences from orthologous loci from both *H. bacteriophora* predictions and a set of 23 rhabditine (Clade V) nematodes (see Supporting Data: *Orthofinder_analysis*) were aligned and analysed with RaxML using a PROTGAMMAGTR amino-acid substitution model. *Pristionchus* spp. were designated as the outgroup. Bootstrap support values (100 bootstraps performed) were 100 for all branches except one.

Tables

. Contamination screening of the *H. bacteriophora* assembly

Number of scaffolds	*Sum of scaffold spans (bp)*	*Mean coverage **	*Best matches in NCBI nt database*	*Assignment*
12	99556	2.8	*Stenotrophomonas maltophilia* genome	bacterial culture contaminant **
4	4709	0.1	*Photorhabdus sp.* genomes	symbiont culture contaminant **
2	2144	756.0	poorly annotated mitochondrial matches	*H. bacteriophora* mitochondrial fragments
22	3051844	69.6	mariner transposons in Metazoa, especially Hymenoptera and Platyhelminthes	*H. bacteriophora* nuclear genome mariner transposon family (highest coverage 960-fold)
10	334100	76.6	low score match to several histone H3.3 across Metazoa	*H. bacteriophora* nuclear sequence
7	713932	56.5	chance nucleotide matches to conserved genes in other taxa	*H. bacteriophora* nuclear sequences

* The average read coverage of the whole assembly was 85.3.

** These scaffolds were removed by the low-coverage filter.

Table 2. Comparison of the published and BRAKER1/soft-masked protein coding gene predictions.

Prediction set	*Published* [14]	*BRAKER1/soft-masked*
Number of protein coding genes predicted	20964	1,5747
Mean protein length (amino acids)	218.8	344.5
Number of single exon genes	1728	2326
Mean number of exons per gene*	5.9	7.8
Proportion of non-canonical (GC-AG) introns	8.87%	0.79%
Percentage mapping to publicly available transcriptome reads		
Sanger ESTs	80.45%	84.26%
Roche 454 reads	37.18%	58.03%
BUSCO score for proteome		
Complete	47.8%	94%
Fragmented	34.7%	4.3%
Number of proteins with no hits in Uniref90	8,962	2,889
Protein singletons in clustering	5442	1112
Conserved, single-copy orthologues**		
Total	2089	2330
Missing	377	141
Expanded	184	84

* Number of exons: number of coding DNA sequence (CDS) entries per gene for BRAKER1 predictions. CDS features, not exons are outputted by AUGUSTUS in GFF files.

** The list of strict one-to-one orthologues was augmented with protein clusters where 75% of species had single copy representatives (“fuzzy-1-to-1” orthologues identified by KinFin).

Supplementary Files

Supplementary file 1: BRAKER1 and JIGSAW annotation pipelines.

Figure illustrating the differences between the BRAKER1 and the Bai et al 2013 JIGSAW prediction methods used for *Heterorhabditis bacteriophora*. PDF file.

Supplementary file 2: Methods Supplementary Note

A note detailing the command lines used in the generation of the BRAKER1 gene predictions, and the associated analysis. PDF file.

Supporting Data

The Supporting Data [24, 62], for this work is described below:

augustus.aa: BRAKER1/soft-masked annotations of Heterorhabditis bacteriophora. The amino acid sequences of the protein predictions in FASTA format.

augustus.gff: BRAKER1/soft-masked annotations of Heterorhabditis bacteriophora. The GFF format file.

augustus.gtf: BRAKER1/soft-masked annotations of Heterorhabditis bacteriophora. The GTF format file.

augustus.hm.aa: BRAKER1/hard-masked annotations of Heterorhabditis bacteriophora. The amino acid sequences of the protein predictions in FASTA format.

augustus.hm.gff: BRAKER1/hard-masked annotations of Heterorhabditis bacteriophora. The GFF format file.

augustus.hm.gtf: BRAKER1/hard-masked annotations of Heterorhabditis bacteriophora. The GTF format file.

Blobtools_coverage_analysis.txt: COV file (raw output from the blobology pipeline) detailing the base/read coverage of the published assembly with reads from the re-sequencing project. Text file.

BRAKER1_annotation_comparisons.txt: Comparison of the BRAKER1/soft-masked and BRAKER1/hard-masked gene predictions from Heterorhabditis bacteriophora. Tab-delimited text file.

Contaminant_scaffolds.txt: A list of the scaffolds/contigs identified by contamination screening and presented in Table 1. Text file.

EST.acc.txt: Accession numbers for the publically available ESTs used for the EST assembly. Text file.

EST.assembled.fas: Assembled ESTs derived from the publicly available ESTs detailed in EST.acc.txt. FASTA .fas format file.

HBACT_BRAKER1_signalPNoTM.txt: Secretome predictions from the BRAKER1/soft-masked predictions. Text file.

HBACT_published_signalPNoTM.txt: Secretome predictions from the published Bai et al. (2013) protein predictions. Text file.

Individual_gene_alignments: Alignments of orthogroups used to build the supermatrix. Directory of aligned sequences in fasta format.

IPR.domain.analysis.txt: Comparative Interproscan statistics. Text file.

kinfin.zip: KinFin analyses from the OrthoFinder analyses of Heterorhabditis bacteriophora predicted proteomes. Zipped archive (42.6 Mb).

Low_coverage_scaffolds.txt: Scaffolds and contigs removed from the Heterorhabditis bacteriophora assembly because of low coverage in the new whole genome sequencing dataset. Text file.

Newick_tree.txt: Phylogenetic analysis output files. NEWICK format text file.

Orthofinder.zip: The OrthoFinder output files. A zipped archive of the three OrthoFinder clustering result files (published H. bacteriophora + 23 species; BRAKER1/soft-masked + 23 species: published + soft-masked + 23 species). Zipped archive (20.9 Mb)

Orthogroup_count_ratios.txt: Table with count of orthogroups at each contribution ratio from the BRAKER1/soft-masked and published proteomes after clustering with 23 other Clade V nematodes. Empty cells denote contribution combinations with no orthogroups. Text file.

Proteomes_in_clustering.txt: A list of the proteomes included in the OrthoFinder analyses. Text file.

Publicly_available_assembly_details.txt: Details of the published, publicly available Heterorhabditis bacteriophora genome assembly re-analysed in this study using BRAKER1. Text file.

Scaffolds_included.txt: Scaffolds and contigs in the Heterorhabditis bacteriophora assembly included in re-annotation and further analysis. Text file.

Secretome.analysis.txt: Secretome statistics for 23 Clade V nematodes. Text file.

Short_BRAKER1_genes_list.txt: List of Heterorhabditis bacteriophora proteins of length <30 amino acids excluded from the OrthoFinder analyses. Text file

Supermatrix.fas: Supermatrix of aligned sequences. FASTA .fas format file.

Figure 1

Click here to download Figure Figure_1.pdf

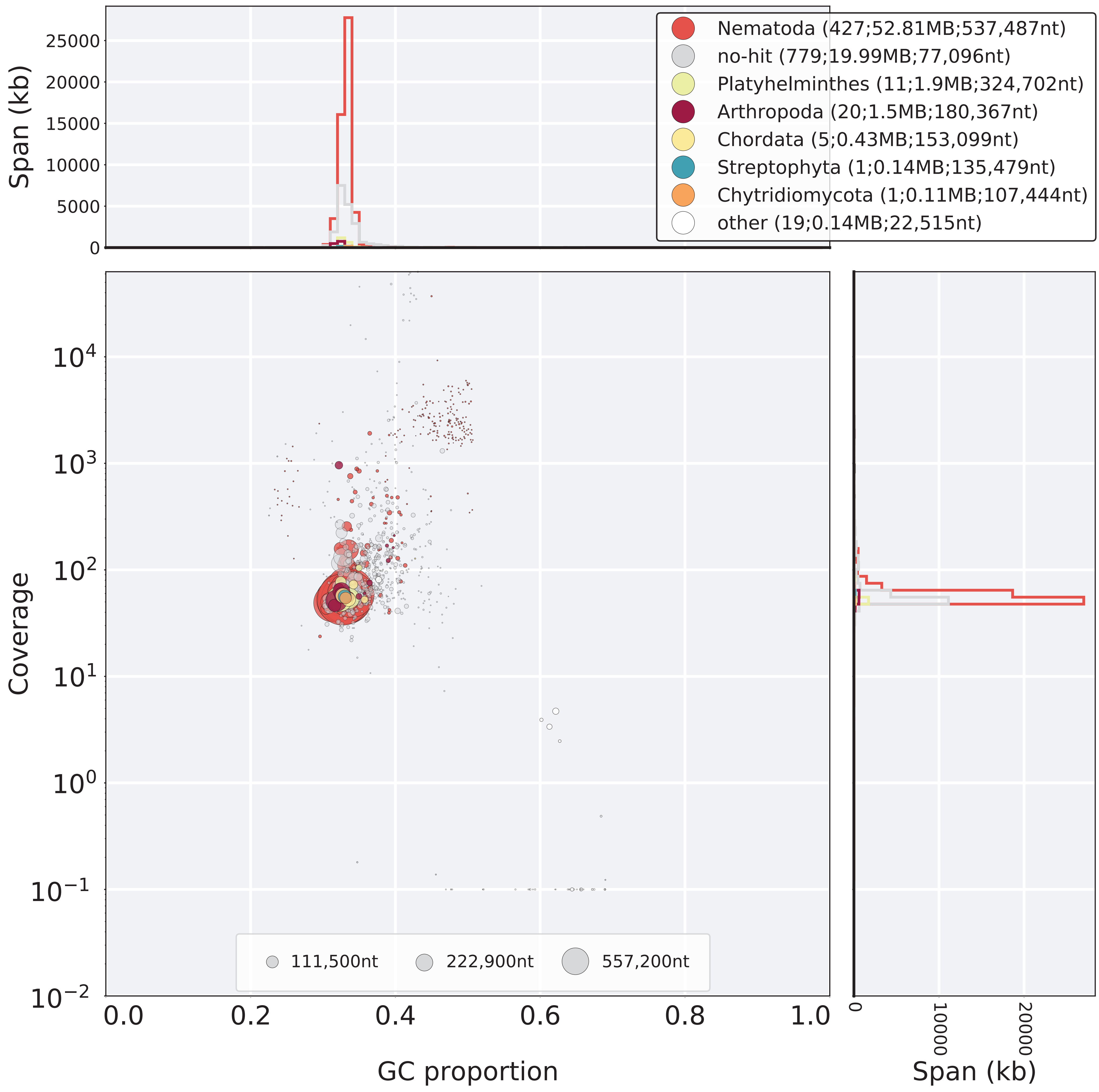

Figure 2 Click here to download Figure Figure_2.pdf

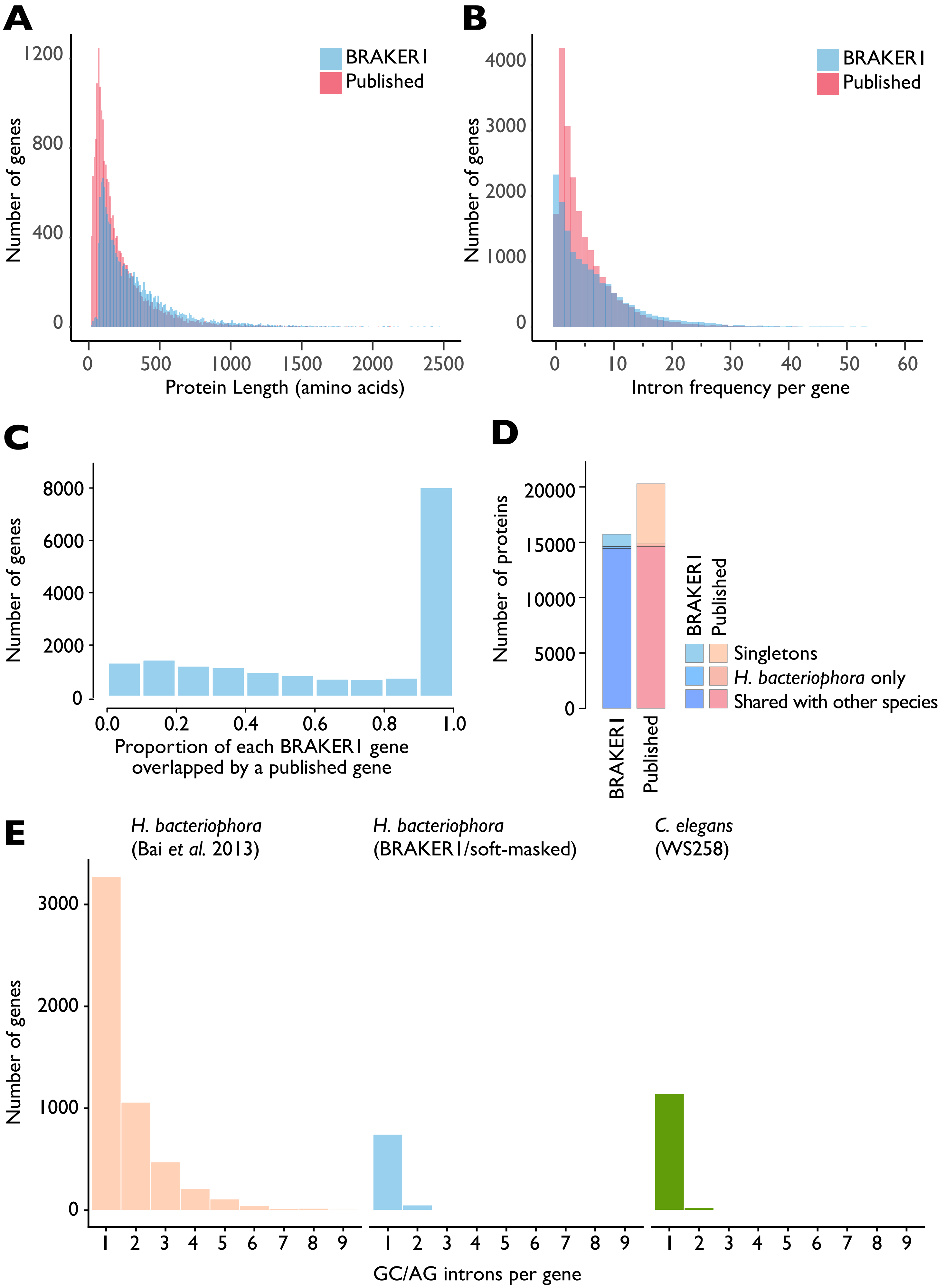

Figure 3 Click here to download Figure Figure_3.pdf

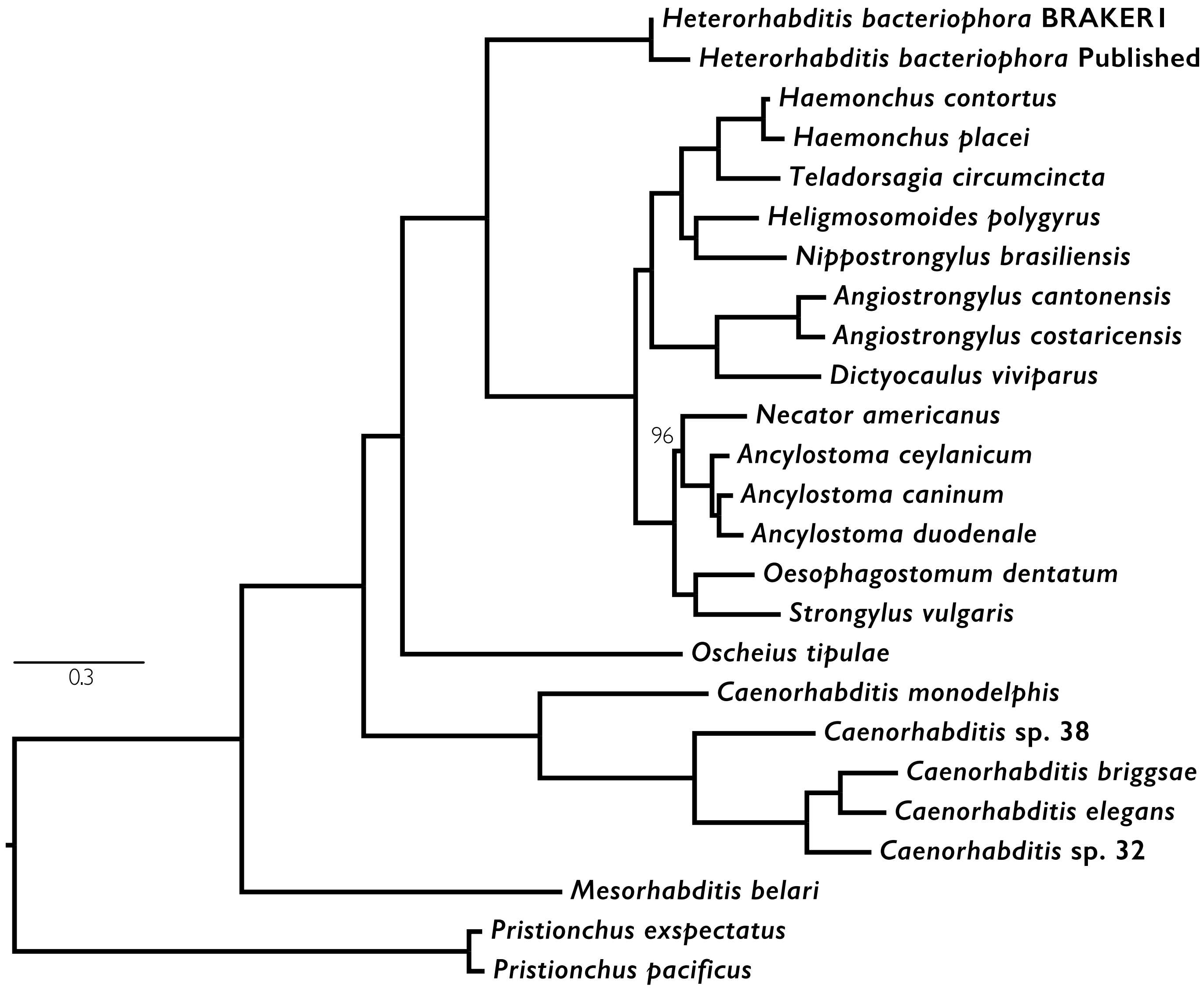

Supplementary File 1

Click here to access/download
Supplementary Material
Supplementary_File_1.pdf

Supplementary File 2

Click here to access/download
Supplementary Material
Supplementary_File_2.docx
