# Supplementary material for: Improving the annotation of the Heterorhabditis bacteriophora genome
Source: Gigascience. 2018 Apr 2;7(4):giy034. doi: 10.1093/gigascience/giy034 (PMC5906903; doi:10.1093/gigascience/giy034)
Supplement: Supplemental material [file giy034_supp.zip › Supplementary_File_1.pdf]

Supplementary File 1. BRAKER1 and JIGSAW annotation pipelines.

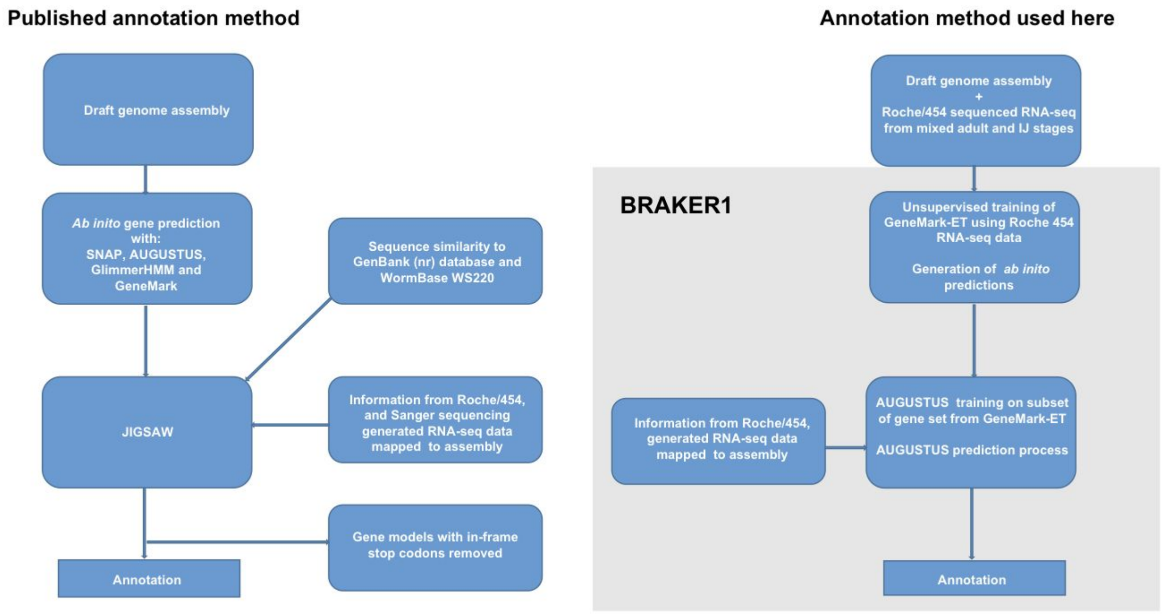

Both methods used masking of the draft assembly before annotation, however the details of masking in the published methods are not explicit [13]. Of note, the published annotation method (left) made use of two transcriptome sets generated by different sequencing methods (Sanger sequencing and Roche 454 sequencing) from a mixture of adult and infective juvenile stages [13]. Only one of these publically available sets (the Roche 454 set) was selected for re-annotation with BRAKER1 as the coverage of those in the Sanger sequencing set was incompatible with the BRAKER1 pipeline (right).
