# Supplementary material for: Improving the annotation of the Heterorhabditis bacteriophora genome
Source: Gigascience. 2018 Apr 2;7(4):giy034. doi: 10.1093/gigascience/giy034 (PMC5906903; doi:10.1093/gigascience/giy034)
Supplement: Supplemental material [file giy034_supp.zip › Supplementary_File_2.docx]

**Methods Supplementary Note**

For input data please see **Input data and data availability** in main manuscript.

**Contaminant screening**

**Nucleotide-Nucleotide BLAST of assembly scaffolds against Genbank nt**

To screen the published assembly for contamination, the assembly scaffolds were aligned to the NCBI nt database, release 204, using Nucleotide-Nucleotide BLAST v2.6.0+ [[1]](http://f1000.com/work/citation?ids=1188822&pre=&suf=&sa=0). *H. bacteriophora* hits were excluded from the search using a list of all *H. bacteriophora* associated gene identifiers downloaded from NCBI GenBank nucleotide database (release 219), here represented by [sequence.gi]. BLAST was executed as:

[path to BLAST v.2.6.0+]/blastn -task megablast -query [assembly.fasta] -db [Genbank nt database release 204] -culling_limit 2 -negative_gilist [sequence.gi] -out [assembly.fasta.blastn] -outfmt '6 qseqid staxids bitscore std sscinames sskingdoms stitle' -evalue 1e-25

**Alignment of genomic re-sequencing project reads to the published assembly**

The Illumina reads from the re-sequencing project ([reads.forwards.fastq.gz] and[reads.reverse.fastq.gz]) were mapped against the assembly using Burrows-Wheeler Aligner (BWA) v0.7.15 [[2]](http://f1000.com/work/citation?ids=48641&pre=&suf=&sa=0), and viewed using Samtools v.1.3.1 [[3]](http://f1000.com/work/citation?ids=48787&pre=&suf=&sa=0) as follows:

1. Index the assembly:

[path to BWA v0.7.15]/bwa index [assembly.fasta]

1. Map the reads and convert the resulting sequence alignment map file to a BAM file:

[path to BWA v0.7.17]/bwa mem -t 32 [assembly.fasta] [reads.forwards.fastq.gz] [reads.reverse.fastq.gz]| [path to Samtools v1.3.1]/samtools view -@ 32 -bS > [assembly.fasta.readmap.bam]

1. View the mapping statistics:

[path to Samtools v.1.3.1]/ samtools flagstat [assembly.fasta.readmap.bam]

**Taxon annotated GC coverage plot generation**

Blobtools v0.9.19 [[4]](http://f1000.com/work/citation?ids=4087662&pre=&suf=&sa=0) was used to create a taxon annotated GC-coverage plot for the published assembly, using the Nucleotide-Nucleotide BLAST results and re-sequencing read mapping results (see above) as follows:

1. Create a blobdb.json file

[path to blobtools]/blobtools.py create -i [assembly.fasta] –b [assembly.fasta.readmap.bam] -t [assembly.fasta.blastn]

1. Create a BlobDb.table:

[path to blobtools]/blobtools.py view -i blobDB.json --hits --rank all > [BlobDb.table]

1. Create the plot:

[path to blobtools]/blobtools.py blobplot -i blobDB.json

**Removal of scaffolds with read coverage of <10x**

A list of scaffolds with coverage of <10x was identified from the .cov output file from Blobtools, and these scaffolds removed from the published assembly using the script fastaqual_select.pl [[5]](http://f1000.com/work/citation?ids=4885750&pre=&suf=&sa=0) as follows:

[path to fastaqual_select.pl]/fastaqual_select.pl-f [assembly.fa] -e [Low.coverage.scaffolds.list]> [assembly.high.cov.fa]

Fastaqual_select.pl is publicly available at: https://github.com/sujaikumar/assemblage/blob/master/fastaqual_select.pl

**Generation of BRAKER1 Gene Predictions**

**Masking the assembly for repeats**

The published assembly was soft-masked for known Nematoda repeats from the RepeatMasker Library v4.0.6 using RepeatMasker v4.0.6 [[6]](http://f1000.com/work/citation?ids=44423&pre=&suf=&sa=0) as follows:

1. Create file containing known Nematoda repeats

[path to repeatmasker-4-0-6]/util/queryRepeatDatabase.pl  -species [nematode] | grep -v "Species:"> [file.containing known.repeats]

1. Mask the assembly for these known nematode repeats

[path to repeatmasker-4-0-6]/RepeatMasker -xsmall -lib [file.containing.known.repeats] -pa 16 [assembly.high.cov.fa]

**Quality and adaptor-trimming of the RNA-seq Roche/454 raw reads**

The two publicly available Roche 454 RNA-seq data files were adaptor and quality-trimmed using BBDuk v36.92, (an unpublished toolkit from Joint Genome Institute, n.d) as follows:

[path to BBDuk v36.92]/bbduk.sh in=[reads.fastq] out=[reads.fastq.clean] ref=[path to bbmap-36.92]/resources/adapters.fa threads=16 ktrim=r k=23 mink=11 hammingdistance=1 tpe tbo qtrim=rl trimq=20 minavgquality=10 minlength=25 qin=33

**Aligning the cleaned RNA-seq Roche/454 reads to the published assembly**

The cleaned reads were mapped to the soft-masked assembly using STAR v2.5 [[7, 8]](http://f1000.com/work/citation?ids=49324,3571270&pre=&pre=&suf=&suf=&sa=0,0) as follows:

1. Generate the genome directory:

[path to STAR_2.5]/STAR --runThreadN 8 --runMode genomeGenerate --genomeDir [output.directory] --genomeFastaFiles [assembly.fa]

1. Align the reads:

[path to STAR_2.5]/STAR --runThreadN 8 --genomeDir [output.directory] --readFilesCommand zcat –readFilesIn [RNA-seq.reads.fq.gz,RNA-seq.reads.2.fq.gz]

**Re- annotation of published assembly using BRAKER1**

The soft-masked assembly was annotated with BRAKER1 v1.9, using the output from STAR (see above), as follows:

[path to BRAKER1 v1.9]/braker.pl --workingdir= [your.working.dir] --species=[Species name] --cores=8 --genome=[masked assembly] --bam=[Aligned.out.bam from STAR]

**Gene Prediction Statistics**

**Longest isoform identification and selection**

The longest isoform for each gene in the BRAKER1 *H. bacteriophora* annotation was identified from the general feature format file as follows:

1. Generate a true GFF3 file

gtf2gff.pl < [annotation.gff] --out=[annotation.gff3] --gff3

Gtf2gff.pl [[9]](http://f1000.com/work/citation?ids=4886715&pre=&suf=&sa=0) is a publically available script supporting Augustus, and is available at http://augustus.gobics.de/binaries/scripts/gtf2gff.pl.

2. Get a list of the longest isoforms gene IDs from the gff3

filter_isoforms_based_on_gff3.py -f [protein.fa]-g [annotation.gff3] -o [longest.isoforms.output.file] -t [file type, WormBase or ENSEMBL ] --fs 0

Filter_isoforms_based_on_gff3.py is part of kinfin [[10]](http://f1000.com/work/citation?ids=4213258&pre=&suf=&sa=0), and is publically available at https://github.com/DRL/kinfin/blob/master/scripts/filter_isoforms_based_on_gff3.py

3. Select the longest isoforms to produce a single longest isoform protein file:

fastaqual_select.pl -i [longest.isoforms.output.file] –f [protein.fa]> [longest.isoforms.protein.fa]

Fastaqual_select.pl is publicly available at: https://github.com/sujaikumar/assemblage/blob/master/fastaqual_select.pl

**Gene Prediction statistics**

Gene-level intron and CDS counts for the BRAKER1 gene predictions (see above) were calculated using unix command line tools as follows:

awk '$3==”[intron or CDS]"' [longest.isoforms.aa] | cut -f 9 | uniq -c | less > [intron.or.CDS.feq.list]

Gene-level intron and exon counts for the published gene predictions were calculated as follows:

1. Add introns to the published annotations GFF3 file using Genometools v1.5.9 [[11]](http://f1000.com/work/citation?ids=4367829&pre=&suf=&sa=0) :

[path to genometools v1.5.9]/bin/gt gff3 -addintrons [annotation.gff]>[annotation.with.introns.gff]

2. Calculate intron or exon frequency:

awk '$3==”[intron or exon]"' [annotation.with.introns.gff] | cut -f 9 | uniq -c | less > [intron.or.exon.freq.list]

**Calculation of protein lengths**

Mean protein lengths were calculated from the amino-acid protein sequence files using unix command line tools as follows:

1. Parse the amino-acid sequences to a single line per protein:

sed -e 's/\(^>.*$\)/#\1#/' [ [longest.isoforms.protein.fa]| tr -d "\r"| tr -d "\n" | sed -e 's/$/#/' | tr "#" "\n" | sed -e '/^$/d' > [longest.isoforms.protein.one.line.fa]

1. Calculate the lengths as follows:

cat [longest.isoforms.protein.one.line.fa] | while read line ; do

currentline=$line

if [[ $currentline == ">"* ]] ; then

echo $currentline

else

echo $currentline | awk '{print length}'

fi

done > [protein.lengths.list]

**Assessment of gene overlap**

Gene features, extracted from the GFF files, were assessed for overlap using bedtools v2.26 [[12]](http://f1000.com/work/citation?ids=48789&pre=&suf=&sa=0) as follows:

1. Extract only gene features from both general feature format files using unix command line tools:

grep -v '#' [annotation.gff] | awk '$3=="gene"'  > [gene.features.list]

1. Identify and count overlapping genes:

[path to bedtools v2.26.0]/bin/intersectBed -s -b [BRAKER1.gene.features.list] -a [published.gene.features.list] > [output.file]

wc -l [output.file]

1. Calculate percentage overlap:

[path to bedtools v2.26.0]/bin/intersectBed -s -wao -a [BRAKER1.gene.features.list] -b [published.gene.features.list]> [overlap.output.file]

 awk '{$20=$19/($5-$4)}1' [overlap.output.file] |cut -f 20 -d ' '> [overlap.percentage.file]

1. Calculate number of genes sharing identical scaffold coordinates:

awk '$4==$13 && $5==$14' [overlap.output.file]|wc -l

**Assessment of identical protein sequence number**

The number of identical proteins shared between the published and BRAKER1 proteomes non-redundant protein fasta files were calculated as follows:

1. Get non-redundant protein sequences using cd-hit v4.6.,1 [[13]](http://f1000.com/work/citation?ids=105558&pre=&suf=&sa=0):

[path to cd-hit-v4.6.1]/cd-hit -c 1.0 -t 1 -i [BRAKER1or published.protein.fa] -o [protein.fa.non-redundant]

1. parse the amino-acid sequences to a single line per protein using unix command line tools:

sed -e 's/\(^>.*$\)/#\1#/' [protein.fa.non-redundant] | tr -d "\r" | tr -d "\n" | sed -e 's/$/#/' | tr "#" "\n" | sed -e '/^$/d'> [protein.fa.non-redundant.single.lines]

1. Concatenate the files, sort the sequences alphabetically, get a unique sequence count, and extract duplicated sequences to get number of identical proteins in the published and new proteome

cat [published.protein.fa.non-redundant.single.lines] [BRAKER1.protein.fa.non-redundant.single.lines]| grep -v '>' | sort | uniq -c |grep  '2' > [identical.protein.seq]

wc –l [identical.shared.protein.seq]

**Analysis of splice sites**

1. Add introns to GFF3s using Genometools v1.5.9 [[11]](http://f1000.com/work/citation?ids=4367829&pre=&suf=&sa=0) :

parallel -j3 '[path to GenomeTools v1.5.9]/bin/gt gff3 -sort -tidy -retainids -fixregionboundaries -addintrons {} > {.}.gt_with_introns.gff3' ::: *.gff3

1. Removing non-GenomeTools introns from *C. elegans* GFF3:

awk '$2=="."' caenorhabditis_elegans.PRJNA13758.WBPS8.annotations.wormbase.gt_with_introns.gff3 > caenorhabditis_elegans.PRJNA13758.WBPS8.annotations.wormbase.gt_with_introns.only_gt_introns.gff3

1. Extract splice sites using extractRegionFromCoordinates.py [[14]](http://f1000.com/work/citation?ids=2329397&pre=&suf=&sa=0) :

extractRegionFromCoordinates.py is available at https://github.com/DRL/GenomeBiology2016_globodera_rostochiensis/blob/master/scripts/extractRegionFromCoordinates.py.

./extractRegionsFromCoordinates.py caenorhabditis_elegans.PRJNA13758.WBPS8.annotations.wormbase.gt_with_introns.only_gt_introns.gff3 caenorhabditis_elegans.PRJNA13758.WBPS8.genomic.fa 0 1 1 0 > caenorhabditis_elegans.PRJNA13758.WBPS8.splice_sites_table.txt

./extractRegionsFromCoordinates.py heterorhabditis_bacteriophora.PRJNA13977.WBPS8.annotations.wormbase.gt_with_introns.gff3 heterorhabditis_bacteriophora.PRJNA13977.WBPS8.genomic.fa 0 1 1 0 > heterorhabditis_bacteriophora.PRJNA13977.WBPS8.splice_site_table.txt

./extractRegionsFromCoordinates.py Heterorhabditis_bacteriophora_M31e_PRJNA13977.gt_with_introns.gff3 Heterorhabditis_bacteriophora_M31e_PRJNA13977.scaffolds.fa 0 1 1 0 > Heterorhabditis_bacteriophora_M31e_PRJNA13977.splice_sites_table.txt

1. Extract IDs of transcripts with GC-AG splice sites:

awk '$9=="GC" && $10=="AG"' caenorhabditis_elegans.PRJNA13758.WBPS8.splice_sites_table.txt | cut -f6 | sed 's/Transcript://g' | grep -v 'CDS' | grep -v 'Pseudo' > caenorhabditis_elegans.PRJNA13758.WBPS8.splice_site_table.txt.GCAG_ids.txt

awk '$9=="GC" && $10=="AG"' heterorhabditis_bacteriophora.PRJNA13977.WBPS8.splice_site_table.txt | cut -f6 | sed 's/transcript://g' > heterorhabditis_bacteriophora.PRJNA13977.WBPS8.splice_site_table.txt.GCAG_ids.txt

awk '$9=="GC" && $10=="AG"' Heterorhabditis_bacteriophora_M31e_PRJNA13977.splice_sites_table.txt | cut -f6 | sed 's/transcript://g' > Heterorhabditis_bacteriophora_M31e_PRJNA13977.splice_sites_table.txt.GCAG_ids.txt

1. Count GC-AG splice sites by IDs of transcripts:

for file in *.txt.GC_ids.txt; do uniq -c $file > $file.counts_per_gene.txt; done

1. Plot using Rscript plot_GCAG_counts.R (Supporting Data: plot_GCAG_counts.R):

plot_GCAG_counts.R is also available at <https://github.com/DRL/mclean2017/blob/master/analysis/splice_sites/plot_GCAG_counts.R>

Rscript plot_GCAG_counts.R

**BUSCO scoring**

BUSCO v2.0.1 [[15]](http://f1000.com/work/citation?ids=706970&pre=&suf=&sa=0), with Eukaryota as the lineage dataset, and *Caenorhabditis* as the species parameter for orthologue finding was applied to both proteomes and the published assembly as follows:

[path to busco-v2.0]/BUSCO.py -i [assembly.fa or protein.fa] -o [output.file] -l [path to busco-v2.0]/DATASETS/eukaryota_odb9/ -m [geno or prot] -sp [caenorhabditis]

**Mapping of RNA-seq reads to the predicted transcriptome**

BWA v0.7.15 [[2]](http://f1000.com/work/citation?ids=48641&pre=&suf=&sa=0) was used to map the RNA-seq datasets to the CDS transcripts from the published and BRAKER1 annotations, and the summary statistics obtained with Samtools v1.3.1 [[3]](http://f1000.com/work/citation?ids=48787&pre=&suf=&sa=0) in flagstat mode as follows:

1. Convert the BRAKER1 annotation true GFF3 file (see above “Longest isoform identification and selection”) into a CDS file using cufflinks v2.2.1 (RRID:SCR_014597) [[16]](http://f1000.com/work/citation?ids=48941&pre=&suf=&sa=0) :

[path to cufflinks v2.2.1]/gffread [annotation.gff3] -g [genome.fa] -x [CDS.fa]

A file containing the CDS sequences for the published annotations is publicly available for at Wormbase Parasite (WBPS8) (https://parasite.wormbase.org/index.html).

2. Index the transcriptome:

[path to bwa v0.7.15]/bwa index [CDS.fa]

3. Map the publically available Roche 454 data and Sanger expressed sequence tags, and generate a file containing the summary statistics:

[path to bwa-0.7.15]/bwa mem -t 32 [CDS.fa] [RNA-seq.fa] | [path to samtools-1.3.1]/samtools view -@ 32 -bS > [output.file]

**Align proteomes to Uniref-90 database**

The published and BRAKER1/soft-masked proteomes were compared to the Uniref90 [[17]](http://f1000.com/work/citation?ids=3303669&pre=&suf=&sa=0) (release 03/2017) database, using DIAMOND v0.9.5 [[18]](http://f1000.com/work/citation?ids=429288&pre=&suf=&sa=0) as follows:

[path to diamond-v0.9.5]/diamond blastp --max-target-seqs 0 -c 1 --threads 16 -q [protein.fa] -d [path to Uniref90 database]/[uniref90 database] –o [output.file] --outfmt [6 qseqid sseqid pident length mismatch gapopen qstart qend sstart send evalue] -e [1e-5]

**Protein orthology analysis**

**Orthologue clustering**

OrthoFinder v1.1.4 [[19]](http://f1000.com/work/citation?ids=1098484&pre=&suf=&sa=0) was used to identify orthologous groups in the proteomes of 23 Clade V nematodes with the addition of either the BRAKER1/soft-masked and published *H. bacteriophora* proteomes separately or simultaneously as follows:

[path to orthofinder]/orthofinder.py –f [directory containing protein.fa files] -t 16

**Proteome functional homology search**

Interproscan v5.19-58.0 [[20]](http://f1000.com/work/citation?ids=2892013&pre=&suf=&sa=0) was used in protein mode to identify matches in the BRAKER1 and published proteomes as follows:

[path to interproscan v5.19-58]/interproscan.sh -T /run/shm/ -i [protein.fa] -d [out.directory] -dp -t p --goterms -appl TIGRFAM-15.0,ProDom-2006.1,SMART-7.1,SignalP-EUK-4.1,PrositePatterns-20.119,PRINTS-42.0,SuperFamily-1.75,Pfam-29.0,PrositeProfiles-20.119 -f TSV

and in the remaining 23 other nematode proteomes as follows:

[path to interproscan v5.19-58]/interproscan.sh -T /run/shm/ -i [protein.fa] -d [out.directory] -dp -t p --goterms -appl SignalP-EUK-4.1 -f TSV

Proteins containing stop codons were removed from the proteomes, and the single longest isoforms selected before analysis.

**Fuzzy 1-to-1 orthologue finding**

Kinfin v0.9 [[10]](http://f1000.com/work/citation?ids=4213258&pre=&suf=&sa=0) was used with default settings to identify true and fuzzy 1-to-1 orthologues as follows:

[path to kinfin v0.9]/kinfin.py -p [speciesIDs.txt]

-g [orthogroups.txt]

-c [speciesclassification.txt]

-o [output.file]

-s [sequenceIDs.txt]

--functional_annotation [functional_annotation.txt]

-a [directory.containing.longest.isoform.fasta.files]

See https://kinfin.readme.io/docs for details of input file requirements.

--functional_annotation takes output from Interproscan, converted to table format, using the Kinfin script ips_to_table.py as follows:

[path to script]/ips_to_table.py -i [Interproscan.output] -o [functional_annotation.txt]

**Generate maximum likelihood gene trees**

Both *H. bacteriophora* proteomes were clustered simultaneously with the 23 Clade V nematode proteomes into orthologous groups using Orthofinder v1.0 as above. The fuzzy 1-to-1 orthologues were extracted, concatenated into orthologue group multi fasta files, and processed using GNU parallel to generate gene trees as follows:

1. Align the proteins within each orthogroup using MAFFT v7.267 [[21]](http://f1000.com/work/citation?ids=387873&pre=&suf=&sa=0) :

[path to mafft-7.267]/bin/mafft --auto --reorder [orthogroup.fa] > [orthogroup.fa.aln]

1. Trim the alignments using Noisy v1.5.12 [[22]](http://f1000.com/work/citation?ids=1965722&pre=&suf=&sa=0) :

[path to Noisy-1.5.12]/noisy --seqtype P [orthogroup.fa.aln]

1. Generate the gene trees using RAxML v8.1.20 [[23]](http://f1000.com/work/citation?ids=326392&pre=&suf=&sa=0) :

[path to RAxML-8.1.20]/raxmlHPC-PTHREADS-SSE3 -m PROTGAMMAAUTO -f a -x 12345 -# 100 -T 2 -p 12345 -s [orthogroup.fa.aln.trimmed] -n [output.file]

1. Prune the gene trees to remove paralogues and trees containing <20 species using PhyloTreePruner v1.0 [[24]](http://f1000.com/work/citation?ids=1241601&pre=&suf=&sa=0) :

java PhyloTreePruner [RAxML.bipartitions.file] 20 [orthogroups.fa.aln.trimmed] 0.5 u

**Generate supermatrix Clade V nematode maximum likelihood tree**

1. Align the pruned orthogroups using MAFFT v7.267 [[21]](http://f1000.com/work/citation?ids=387873&pre=&suf=&sa=0) :

[path to mafft-7.267]/bin/mafft --auto --reorder [orthogroup.fa.pruned] > [orthogroup.fa.pruned.aln]

1. Trim the alignment using Noisy v1.5.12 [[22]](http://f1000.com/work/citation?ids=1965722&pre=&suf=&sa=0) :

[path to Noisy-1.5.12]/noisy --seqtype P [orthogroup.fa.aln]

1. From within the directory containing the trimmed alignments, concatenate the orthogroups to make a supermatrix using FASconCAT_v1.0.pl  [[25]](http://f1000.com/work/citation?ids=3690746&pre=&suf=&sa=0) :

[path to FASconCAT_v1.0]/FASconCAT_v1.0.pl -s  -p –n

1. Generate the supermatrix tree using RAxML-8.1.20 [[23]](http://f1000.com/work/citation?ids=326392&pre=&suf=&sa=0) :

[path to RAxML-8.1.20]/raxmlHPC-PTHREADS-SSE3 -m PROTGAMMAAUTO -f a

-x 12345 -# 100 -T 2 -p 12345 -s [supermatrix.fa] \

-n [output.file]

References

[1. Boratyn GM, Camacho C, Cooper PS, Coulouris G, Fong A, Ma N, et al. BLAST: a more efficient report with usability improvements. Nucleic Acids Res. 2013;41 Web Server issue:W29-33. doi:10.1093/nar/gkt282.](http://f1000.com/work/bibliography/1188822)

[2. Li H, Durbin R. Fast and accurate short read alignment with Burrows-Wheeler transform. Bioinformatics. 2009;25:1754–60. doi:10.1093/bioinformatics/btp324.](http://f1000.com/work/bibliography/48641)

[3. Li H, Handsaker B, Wysoker A, Fennell T, Ruan J, Homer N, et al. The Sequence Alignment/Map format and SAMtools. Bioinformatics. 2009;25:2078–9. doi:10.1093/bioinformatics/btp352.](http://f1000.com/work/bibliography/48787)

[4. Laetsch DR, Blaxter ML. BlobTools: Interrogation of genome assemblies [version 1; referees: 2 approved with reservations]. F1000Res. 2017;6:1287. doi:10.12688/f1000research.12232.1.](http://f1000.com/work/bibliography/4087662)

[5. Kumar S. fastaqual_select.pl. 2012. https://github.com/sujaikumar/assemblage/blob/master/fastaqual_select.pl. Accessed 22 Feb 2018.](http://f1000.com/work/bibliography/4885750)

[6. Tarailo-Graovac M, Chen N. Using RepeatMasker to identify repetitive elements in genomic sequences. Curr Protoc Bioinformatics. 2009;Chapter 4:Unit 4.10. doi:10.1002/0471250953.bi0410s25.](http://f1000.com/work/bibliography/44423)

[7. Dobin A, Davis CA, Schlesinger F, Drenkow J, Zaleski C, Jha S, et al. STAR: ultrafast universal RNA-seq aligner. Bioinformatics. 2013;29:15–21. doi:10.1093/bioinformatics/bts635.](http://f1000.com/work/bibliography/49324)

[8. Dobin A, Gingeras TR. Optimizing RNA-Seq Mapping with STAR. Methods Mol Biol. 2016;1415:245–62. doi:10.1007/978-1-4939-3572-7_13.](http://f1000.com/work/bibliography/3571270)

[9. Stanke M. gtf2gff.pl. 2010. http://augustus.gobics.de/binaries/scripts/gtf2gff.pl. Accessed 23 Feb 2018.](http://f1000.com/work/bibliography/4886715)

[10. Laetsch DR, Blaxter ML. KinFin: Software for Taxon-Aware Analysis of Clustered Protein Sequences. G3 (Bethesda). 2017;7:3349–57. doi:10.1534/g3.117.300233.](http://f1000.com/work/bibliography/4213258)

[11. Gremme G, Steinbiss S, Kurtz S. GenomeTools: a comprehensive software library for efficient processing of structured genome annotations. IEEE/ACM Trans Comput Biol Bioinform. 2013;10:645–56. doi:10.1109/TCBB.2013.68.](http://f1000.com/work/bibliography/4367829)

[12. Quinlan AR, Hall IM. BEDTools: a flexible suite of utilities for comparing genomic features. Bioinformatics. 2010;26:841–2. doi:10.1093/bioinformatics/btq033.](http://f1000.com/work/bibliography/48789)

[13. Huang Y, Niu B, Gao Y, Fu L, Li W. CD-HIT Suite: a web server for clustering and comparing biological sequences. Bioinformatics. 2010;26:680–2. doi:10.1093/bioinformatics/btq003.](http://f1000.com/work/bibliography/105558)

[14. Eves-van den Akker S, Laetsch DR, Thorpe P, Lilley CJ, Danchin EGJ, Da Rocha M, et al. The genome of the yellow potato cyst nematode, Globodera rostochiensis, reveals insights into the basis of parasitism and virulence. Genome Biol. 2016;17:124. doi:10.1186/s13059-016-0985-1.](http://f1000.com/work/bibliography/2329397)

[15. Simão FA, Waterhouse RM, Ioannidis P, Kriventseva EV, Zdobnov EM. BUSCO: assessing genome assembly and annotation completeness with single-copy orthologs. Bioinformatics. 2015;31:3210–2. doi:10.1093/bioinformatics/btv351.](http://f1000.com/work/bibliography/706970)

[16. Trapnell C, Roberts A, Goff L, Pertea G, Kim D, Kelley DR, et al. Differential gene and transcript expression analysis of RNA-seq experiments with TopHat and Cufflinks. Nat Protoc. 2012;7:562–78. doi:10.1038/nprot.2012.016.](http://f1000.com/work/bibliography/48941)

[17. Boutet E, Lieberherr D, Tognolli M, Schneider M, Bansal P, Bridge AJ, et al. UniProtKB/Swiss-Prot, the Manually Annotated Section of the UniProt KnowledgeBase: How to Use the Entry View. Methods Mol Biol. 2016;1374:23–54. doi:10.1007/978-1-4939-3167-5_2.](http://f1000.com/work/bibliography/3303669)

[18. Buchfink B, Xie C, Huson DH. Fast and sensitive protein alignment using DIAMOND. Nat Methods. 2015;12:59–60. doi:10.1038/nmeth.3176.](http://f1000.com/work/bibliography/429288)

[19. Emms DM, Kelly S. OrthoFinder: solving fundamental biases in whole genome comparisons dramatically improves orthogroup inference accuracy. Genome Biol. 2015;16:157. doi:10.1186/s13059-015-0721-2.](http://f1000.com/work/bibliography/1098484)

[20. Finn RD, Attwood TK, Babbitt PC, Bateman A, Bork P, Bridge AJ, et al. InterPro in 2017-beyond protein family and domain annotations. Nucleic Acids Res. 2017;45:D190–9. doi:10.1093/nar/gkw1107.](http://f1000.com/work/bibliography/2892013)

[21. Katoh K, Standley DM. MAFFT multiple sequence alignment software version 7: improvements in performance and usability. Mol Biol Evol. 2013;30:772–80. doi:10.1093/molbev/mst010.](http://f1000.com/work/bibliography/387873)

[22. Dress AWM, Flamm C, Fritzsch G, Grünewald S, Kruspe M, Prohaska SJ, et al. Noisy: identification of problematic columns in multiple sequence alignments. Algorithms Mol Biol. 2008;3:7. doi:10.1186/1748-7188-3-7.](http://f1000.com/work/bibliography/1965722)

[23. Stamatakis A. RAxML-VI-HPC: maximum likelihood-based phylogenetic analyses with thousands of taxa and mixed models. Bioinformatics. 2006;22:2688–90. doi:10.1093/bioinformatics/btl446.](http://f1000.com/work/bibliography/326392)

[24. Kocot KM, Citarella MR, Moroz LL, Halanych KM. PhyloTreePruner: A Phylogenetic Tree-Based Approach for Selection of Orthologous Sequences for Phylogenomics. Evol Bioinform Online. 2013;9:429–35. doi:10.4137/EBO.S12813.](http://f1000.com/work/bibliography/1241601)

[25. Kück P, Meusemann K. FASconCAT: Convenient handling of data matrices. Mol Phylogenet Evol. 2010;56:1115–8. doi:10.1016/j.ympev.2010.04.024.](http://f1000.com/work/bibliography/3690746)
