# Supplementary material for: Improving the annotation of the Heterorhabditis bacteriophora genome
Source: Gigascience. 2018 Apr 2;7(4):giy034. doi: 10.1093/gigascience/giy034 (PMC5906903; doi:10.1093/gigascience/giy034)
Supplement: Response_to_Reviewer_Comments_Original_Submission.pdf [file giy034_response_to_reviewer_comments_original_submission.pdf]

Author's Response To Reviewer Comments

00000000-0000-(9ged59Y3KzVPH

E2A9317A Z/VDZKqWlt+UU|

Close

Date 05.03.2018

Dear GigaScience Editors,

Re: Resubmission of manuscript: Improving the annotation of the Heterorhabditis bacteriophora genome

Thank you for the opportunity to revise our manuscript, Improving the annotation of the Heterorhabditis bacteriophora genome. After reviewing the GigaScience Article Types instructions we are re-submitting the manuscript for consideration for publication as a Data Note as we agree that it fits best as such.

We are grateful for the constructive and positive suggestions from all of the reviewers, as well as their attention to detail. In particular, we have added a supplementary document detailing the command lines used to carry out the analysis, and hope that this will prove useful to those wishing to replicate the experiments.

Below are the comments with our responses indicated. The accompanying manuscript has the corresponding corrections and changes.

Yours Sincerely,

Florence McLean

Reviewer #1:

This manuscript describes the reannotation of the Heterorhabditis bacteriophora, an entomopathogenic nematode widely used to control insect pests in horticulture.
A previous study was reported to encode an unusually high proportion of unique proteins and a paucity of secreted proteins compared to other related nematodes. This study asked whether these unusual characteristics were biological or methodological in origin.

The work was carried out in the spirit of data improvement, rather than a rebuttal, and while it is not a genome paper as such, it does reanalyse a genome using new data and different tools. It is very suited to the GigaScience philosophy and readership due to the repeatable side and open access component.

I have checked that the Methods described and the Resources used meet the minimum standards reporting check list. I note that data has been submitted to the publicly available repositories (SRA and INSDC) but that the data is not yet available, thus it cannot be reviewed at the moment.

**********Response*******************
The reads from the re-sequencing project are still in the process of being submitted to the SRA and the DOI will be advised as soon as it is obtained.

Submission of the revised annotations to INSDC has been delayed over a question of where they would fit into the ENA's data structure. The GFF file has therefore been submitted to Zenodo (DOI:10.5281/zenodo.1169646), and is included in the supplementary data uploaded to the GigaScience DB.
****

I have looked at the files in https://github.com/DRL/mclean2017
There are 9 supplementary files of annotation, analyses and annotation pipelines which look thorough and complete.

The repository also include splice site files.

The manuscript states that all custom scripts developed for this manuscript are available at in this repository but I see only a single script in the /analysis folder. Is this right?

**********Response******************
Very few custom scripts were developed for the analysis of the data, the bulk of which was carried out by executing published programs on the command line, and most basic statistics reported in the manuscript (such as counts) were obtained from manipulation and interrogation of files using unix command line tools. Although these processes were not developed as scripts, we strongly agree with both reviewer #1 here, and reviewer #3 (see below), that provision of the code used in the analysis would greatly enhance the manuscript. We have added a Methods Supplementary Note (Supplementary File 2) to this effect.
****

The gene prediction and protein orthology analyses and discussion were thorough and fully explained, as well as future work (expanded transcriptome and comparative data work) described.

My recommendation is that this manuscript be published as a research article.

I have some minor typos and suggestions which are probably more pertinent for a copy editor to spot but include them here since I noted them down.

105 BUSCO; see below). Another unusual feature of the H. bacteriophora gene set was the ->
105 BUSCO; see Table 2). Another unusual feature of the H. bacteriophora gene set was the

**********Response******************
Corrected
****

107 Most nematode (and other metazoan) genomes have low proportions of non-canonical introns (less than 1%),
[Reference needed]

**********Response******************
Reference provided
****

137 from the new Illumina data and sequence similarity from the NCBI nucleotide database (nt) ->
137 from the new Illumina data and sequence similarity from the NCBI nucleotide (nt) database

**********Response******************
Corrected
****

371 The assembly scaffolds were aligned to the NCBI nucleotide (nt) database, ->
371 The assembly scaffolds were aligned to the NCBI nt database,

**********Response******************
Corrected
****

397 version of the assembly. Hard masking was for known Nematoda repeats from the ->
397 version of the assembly. The assembly was hard-masked for known Nematoda repeats from the….?

**********Response******************
Corrected
****

[Hard masked / hard-masked
Soft masked / soft-masked
check for consistent use]

**********Response******************
Corrected to consistent use of hyphen

****

406 bacteriophora annotation was identified from the general feature format file, and then->
406 bacteriophora annotation was identified from the general feature format (GFF) file, and then

**********Response******************
Corrected
****

407 selected from the protein FASTA files. The general feature format file (GFF) for ->
407 selected from the protein FASTA files. The GFF file for

**********Response******************
Corrected
****

415 from the general feature format file as exon features ->
415 from the GFF file as exon features

**********Response******************
Corrected
****

423 bacteriophora. Intronic features were added to GFF3
[Explain what GFF3 is]

Expanded to general feature format version 3 (GFF3)

[Check consistent use of GFF (line 415) / GFF file / GFF format (744, 749)
Should be GFF file]

**********Response******************
Corrected to be consistent
****

424 gff3 -sort -tidy -retainids -fixregionboundaries -addintrons') and and splice sites were ->
424 gff3 -sort -tidy -retainids -fixregionboundaries -addintrons') and splice sites were

**********Response******************
Corrected
****

445 the 23 Clade V nematodes were downloaded from WBPS8 (available at:
446 http://parasite.wormbase.org/index.html)
[Suggest link to ftp://ftp.ebi.ac.uk/pub/databases/wormbase/parasite/releases/WBPS8/)

**********Response******************
Link changed to that suggested
****

358 Parasite (WBPS8) [34].
[This is the first mention of WormBase Parasite so should include the home page rather than line in 446]

**********Response******************
Suggested link inserted into formerly line [358] and removed from formerly line [446]
****

478 using MAFFT v7.267 (RRID:SCR_011811) [50], and the alignments trimmed with NOISY
[Reference needed for NOISY.]

**********Response******************
Reference added
****

480 v8.1.20 (RRID:SCR_006086) [51] with a PROTGAMMAGTR

[Reference needed for PROTGAMMAGTR]

**********Response******************
Reference provided for RAXML. PROTGAMMAGTR is an option used within RAXML.
****

Reviewer #2:

The manuscript "Improving the annotation of the Heterorhabditis bacteriophora genome" presents the re-annotation of an existing high-quality genome assembly which previously had low-quality gene annotation with many issues. By utilizing RNA-Seq datasets and using the latest high-quality annotation tool (BRAKER1), significant improvements were made in completeness, unique protein counts and secretion predictions. This annotation improvement represents a very significant improvement in how results from Heterorhabditis bacteriophora genome studies will be interpreted.

- The supporting data files are thorough and complete, and support the findings. One suggestion: Although not part of the study, a text file could be added within Supp Tables 2 and 3 which provides the WormBase assembly version used, and accession IDs / web links to the genome assembly, so that readers can have all the information they need to work with the new annotation within the single files.

**********Response******************
Many thanks for your suggestion. I have added a text file into the Supporting data called Publicly_available_assembly_details.txt which details the source, provider, WormBase assembly version used, its Bioproject ID, and the FTP address for easy download.
****

- Tables 1 and 2 in the main text should also be reformatted. Shading is not permitted by Gigascience. Also, removing vertical lines (both tables) and centering the numbers on table 1 would help to improve their look.

**********Response******************
Done- many thanks for the feedback
****

- Please ensure that SRA and INSDC accessions are added, since they are currently referenced as "XXXXXXX"

**********Response******************
Please see comment above to reviewer # 1
****

- Since InterProScan was ran, it would be interesting to look at the statistics in regards to the identification of InterPro domains. For example, compare the number of proteins with any annotated IPR domains, the total number of IPR domains identified, and the number of unique IPR domains identified. The previous publication also performed this comparison with other species using KEGG, so it may be interesting to repeat that similar analysis with the current annotation, although there are many updated ways to run KEGG so the re-analysis of the previous annotation may not match what was previously found.

**********Response******************
Thank you for this suggestion. Extraction of these interproscan statistics did provide further encouraging results. We have included a Supporting data file called IPR.domain.analysis.txt containing the suggested Interproscan statistics and a paragraph has been added to the text to describe the results. We do not feel that the original Kegg analysis in the published paper generated meaningful biological insights and have therefore not replicated it here.
****

Reviewer #3:

Dear authors,

thank you for publishing the re-annotation of Heterorhabditis bacteriophora. It is both interesting for the particular research community dealing with Heterorhabditis bacteriophora, as well as for all research communities dealing with non-model organisms, in general. You demonstrate that the software applied for annotating a species can heavily impact conclusions drawn from a genome annotation project; and

that it is worth re-annotating also non-model organisms with state of the art tools.

Below, you find my review, structured according to the Guide for GigaScience reviewers.

1. Is the rationale for collecting and analyzing the data well defined?

Yes.

2. Is it clear how data was collected and curated?

Yes, it is very clear.

3. Is it clear - and was a statement provided - on how data and analyses tools used in the study can be accessed?

For data, it is very clear.

The authors also make an effort to demonstrate tool availability (not their own, but software developed by others) by providing RRIDs. However, in some cases, the provided RRIDs are more confusing than helpful.

RRID:SCR_008419 is given for BLAST v2.6.0+ but the RRID leads to an URL that is not available (and in the past, when it was available, it corresponded to a particular BLAST interface for balsting against Aedes aegypti, an organsim that is not relevant to the manuscript under review). In this case, it would be more helpful to provide e.g. an URL to the download location of BLAST v2.6.0+; or create a new RRID.

**********Response*******************
Apologies for this error, thank you for noting it. A URL for downloading BLAST v2.6.0 has been provided and the incorrect RRID removed.
****

RRID:SCR_005622 is given for the RNA-Seq aligner STAR; the RRID leads to an URL for a user/password protected STAR related web application. I strongly assume the authors ran STAR locally, and thus, an URL to the offical STAR website would be more appropriate (https://github.com/alexdobin/STAR/releases), or the creation of a new RRID.

**********Response*******************
RRID corrected to the official website version

****
For Rstudio, accidentally, the RRID to STAR web application is provided. Please update to correct RRID or URL.

**********Response*******************
Corrected- thank you again for noting this error.
****

(No RRID or URL is provided for BRAKER. The URL is available in the referenced manuscript, though, and I believe that is sufficient. However, if journal policy is to always print RRIDs or URLs, you might want to add one of the download URLs. Also, BRAKER1 is the only tool where to do not list the version number (braker.pl --version).)

Version added.

4. Are accession numbers given or links provided for data that, as a standard, should be submitted to a community approved public repository?

In principle, yes, some accession numbers were still missing during the review process but will be updated by the authors prior publication.

5. Is the data and software available in the public domain under a Creative Commons license?

Scripts implemented particularly for this publication are avaiable at github, the license is GNU Public License V3. There are differences between licenses, I kindly ask the journal to check whether GPL fulfills

the journal's requirements.

6. Are the data sound and well controlled?

Yes.

7. Is the interpretation (Analysis and Discussion) well balanced and supported by the data?

Yes.

8. Are the methods appropriate, well described, and include sufficient details and supporting information to allow others to evaluate and replicate the work?

In principle: yes. However, it might be useful to the community to provide not only references to the particular tool and version, but also the exact command lines that were used in this project. It would be really nice if you added the command lines to some supplementary document. For example, a reader who knows that BRAKER1 software, will assume that braker was called with the option --softmasking when the authors state that it was applied to a softmasked genome. A reader who is less familiar with the software will maybe not know this and might thus not be able to replicate the experiments, exactly.

**********Response*******************
Thank you for this suggestion, we agree and a Supplementary note has been added to this effect.
****

9. What are the strengths and weaknesses of the methods?

The authors used state of the art methods in a very suitable way.

10. Have the authors followed best-practices in reporting standards?

Yes.

11. Can the writing, organization, tables and figures be improved?

I am not a native speaker of English, myself, but I believe the language is good.

I hope that 1.747 as number of protein coding genes predicted by BRAKER1/soft-masked in Table 2 is a typo, please fix.

**********Response*******************
Corrected- thank you for spotting this
****

12. When revisions are requested.

Minor revisions:

Please correct used software accessiblity references as recommended in point 3.- corrected as above

Please correct typo in Table 2 (point 11).- corrected as above.

Discretionary revisions:

Please consider my statement to point 8.- corrected as above

The journal should probably have a look at the license issue (point 5).

13. Are there any ethical or competing interests issues you would like to raise?

No.

I hope you find this review useful.

Kind regards,

Katharina Hoff

Close
