# Supplementary material for: Improving the annotation of the Heterorhabditis bacteriophora genome
Source: Gigascience. 2018 Apr 2;7(4):giy034. doi: 10.1093/gigascience/giy034 (PMC5906903; doi:10.1093/gigascience/giy034)
Supplement: Reviewer_1_Report_(Original_Submission) -- Mary Ann Tuli — 12/13/2017 Reviewed [file giy034_reviewer_1_report_(original_submission).pdf]

Reviewer Report

Title: Improving the annotation of the Heterorhabditis bacteriophora genome

Version: Original Submission **Date:** 12/13/2017

Reviewer name: Mary Ann Tuli

Reviewer Comments to Author:

This manuscript describes the reannotation of the Heterorhabditis bacteriophora, an entomopathogenic nematode widely used to control insect pests in horticulture.
A previous study was reported to encode an unusually high proportion of unique proteins and a paucity of secreted proteins compared to other related nematodes. This study asked whether these unusual characteristics were biological or methodological in origin.

The work was carried out in the spirit of data improvement, rather than a rebuttal, and while it is not a genome paper as such, it does reanalyse
a genome using new data and different tools. It is very suited to the GigaScience philosophy and readership due to the repeatable side and open access component.

I have checked that the Methods described and the Resources used meet the minimum standards reporting check list. I note that data has been submitted to the publicly available repositories (SRA and INSDC) but that the data is not yet available, thus it cannot be reviewed at the moment.

I have looked at the files in https://github.com/DRL/mclean2017
There are 9 supplementary files of annotation, analyses and annotation pipelines which look thorough and complete.
The repository also include splice site files.
The manuscript states that all custom scripts developed for this manuscript are available at in this repository but I see only a single script in the /analysis folder. Is this right?

The gene prediction and protein orthology analyses and discussion were thorough and fully explained, as well as future work (expanded transcriptome and comparative data work) described.

My recommendation is that this manuscript be published as a research article.

I have some minor typos and suggestions which are probably more pertinent for a copy editor to spot but include them here since I noted them down.

105 BUSCO; see below). Another unusual feature of the H. bacteriophora gene set was the ->
105 BUSCO; see Table 2). Another unusual feature of the H. bacteriophora gene set was the

107 Most nematode (and other metazoan) genomes have low proportions of non-canonical introns (less than 1%),
[Reference needed]

137 from the new Illumina data and sequence similarity from the NCBI nucleotide database (nt) ->
137 from the new Illumina data and sequence similarity from the NCBI nucleotide (nt) database

371 The assembly scaffolds were aligned to the NCBI nucleotide (nt) database, ->
371 The assembly scaffolds were aligned to the NCBI nt database,

397 version of the assembly. Hard masking was for known Nematoda repeats from the ->
397 version of the assembly. The assembly was hard-masked for known Nematoda repeats from the....?

[Hard masked / hard-masked
Soft masked / soft-masked
check for consistent use]

406 bacteriophora annotation was identified from the general feature format file, and then->
406 bacteriophora annotation was identified from the general feature format (GFF) file, and then

407 selected from the protein FASTA files. The general feature format file (GFF) for ->
407 selected from the protein FASTA files. The GFF file for

415 from the general feature format file as exon features ->
415 from the GFF file as exon features

423 bacteriophora. Intronic features were added to GFF3
[Explain what GFF3 is]

[Check consistent use of GFF (line 415) / GFF file / GFF format (744, 749)
Should be GFF file]

424 gff3 -sort -tidy -retainids -fixregionboundaries -addintrons') and and splice sites were ->
424 gff3 -sort -tidy -retainids -fixregionboundaries -addintrons') and splice sites were

445 the 23 Clade V nematodes were downloaded from WBPS8 (available at:
446 http://parasite.wormbase.org/index.html)
[Suggest link to ftp://ftp.ebi.ac.uk/pub/databases/wormbase/parasite/releases/WBPS8/)

358 Parasite (WBPS8) [34].
[This is the first mention of WormBase Parasite so should include the home page rather than line in 446]

478 using MAFFT v7.267 (RRID:SCR_011811) [50], and the alignments trimmed with NOISY
[Reference needed for NOISY.]

480 v8.1.20 (RRID:SCR_006086) [51] with a PROTGAMMAGTR
[Reference needed for PROTGAMMAGTR]

Methods

Are the methods appropriate to the aims of the study, are they well described, and are necessary controls included? Yes

Conclusions

Are the conclusions adequately supported by the data shown? Yes

Reporting Standards

Does the manuscript adhere to the journal’s guidelines on minimum standards of reporting? Yes

Choose an item.

Statistics

Are you able to assess all statistics in the manuscript, including the appropriateness of statistical tests used? Yes, and I have assessed the statistics in my report.

Quality of Written English

Please indicate the quality of language in the manuscript: Acceptable

Declaration of Competing Interests

Please complete a declaration of competing interests, considering the following questions:

- Have you in the past five years received reimbursements, fees, funding, or salary from an organisation that may in any way gain or lose financially from the publication of this manuscript, either now or in the future?
- Do you hold any stocks or shares in an organisation that may in any way gain or lose financially from the publication of this manuscript, either now or in the future?
- Do you hold or are you currently applying for any patents relating to the content of the manuscript?
- Have you received reimbursements, fees, funding, or salary from an organization that holds or has applied for patents relating to the content of the manuscript?
- Do you have any other financial competing interests?
- Do you have any non-financial competing interests in relation to this paper?

If you can answer no to all of the above, write 'I declare that I have no competing interests' below. If your reply is yes to any, please give details below.

I declare that I have no competing interests.

I agree to the open peer review policy of the journal. I understand that my name will be included on my report to the authors and, if the manuscript is accepted for publication, my named report including any attachments I upload will be posted on the website along with the authors' responses. I agree for my report to be made available under an Open Access Creative Commons CC-BY license (http://creativecommons.org/licenses/by/4.0/). I understand that any comments which I do not wish to be included in my named report can be included as confidential comments to the editors, which will not be published.

I agree to the open peer review policy of the journal

To further support our reviewers, we have joined with Publons, where you can gain additional credit to further highlight your hard work (see: https://publons.com/journal/530/gigascience). On publication of this paper, your review will be automatically added to Publons, you can then choose whether or not to claim your Publons credit. I understand this statement.

Yes Choose an item.
