# Supplementary material for: Improving the annotation of the Heterorhabditis bacteriophora genome
Source: Gigascience. 2018 Apr 2;7(4):giy034. doi: 10.1093/gigascience/giy034 (PMC5906903; doi:10.1093/gigascience/giy034)
Supplement: Reviewer_2_Report_(Original_Submission) -- Bruce A Rosa — 12/15/2017 Reviewed [file giy034_reviewer_2_report_(original_submission).pdf]

Reviewer Report

Title: Improving the annotation of the Heterorhabditis bacteriophora genome

Version: Original Submission **Date:** 12/15/2017

Reviewer name: Bruce A Rosa

Reviewer Comments to Author:

The manuscript "Improving the annotation of the Heterorhabditis bacteriophora genome" presents the re-annotation of an existing high-quality genome assembly which previously had low-quality gene annotation with many issues. By utilizing RNA-Seq datasets and using the latest high-quality annotation tool (BRAKER1), significant improvements were made in completeness, unique protein counts and secretion predictions. This annotation improvement represents a very significant improvement in how results from Heterorhabditis bacteriophora genome studies will be interpreted.

- The supporting data files are thorough and complete, and support the findings. One suggestion: Although not part of the study, a text file could be added within Supp Tables 2 and 3 which provides the WormBase assembly version used, and accession IDs / web links to the genome assembly, so that readers can have all the information they need to work with the new annotation within the single files.

- Tables 1 and 2 in the main text should also be reformatted. Shading is not permitted by Gigascience. Also, removing vertical lines (both tables) and centering the numbers on table 1 would help to improve their look.

- Please ensure that SRA and INSDC accessions are added, since they are currently referenced as "XXXXXXX"

- Since InterProScan was ran, it would be interesting to look at the statistics in regards to the identification of InterPro domains. For example, compare the number of proteins with any annotated IPR domains, the total number of IPR domains identified, and the number of unique IPR domains identified. The previous publication also performed this comparison with other species using KEGG, so it may be interesting to repeat that similar analysis with the current annotation, although there are many updated ways to run KEGG so the re-analysis of the previous annotation may not match wha was previously found.

Methods

Are the methods appropriate to the aims of the study, are they well described, and are necessary controls included? Yes

Conclusions

Are the conclusions adequately supported by the data shown? Yes

Reporting Standards

Does the manuscript adhere to the journal's guidelines on [minimum standards of reporting?]() Yes

Choose an item.

Statistics

Are you able to assess all statistics in the manuscript, including the appropriateness of statistical tests used? Yes, and I have assessed the statistics in my report.

Quality of Written English

Please indicate the quality of language in the manuscript: Acceptable

Declaration of Competing Interests

Please complete a declaration of competing interests, considering the following questions:

- Have you in the past five years received reimbursements, fees, funding, or salary from an organisation that may in any way gain or lose financially from the publication of this manuscript, either now or in the future?
- Do you hold any stocks or shares in an organisation that may in any way gain or lose financially from the publication of this manuscript, either now or in the future?
- Do you hold or are you currently applying for any patents relating to the content of the manuscript?
- Have you received reimbursements, fees, funding, or salary from an organization that holds or has applied for patents relating to the content of the manuscript?
- Do you have any other financial competing interests?
- Do you have any non-financial competing interests in relation to this paper?

If you can answer no to all of the above, write 'I declare that I have no competing interests' below. If your reply is yes to any, please give details below.

I declare that I have no competing interests

I agree to the open peer review policy of the journal. I understand that my name will be included on my report to the authors and, if the manuscript is accepted for publication, my named report including any attachments I upload will be posted on the website along with the authors' responses. I agree for my report to be made available under an Open Access Creative Commons CC-BY license (http://creativecommons.org/licenses/by/4.0/). I understand that any comments which I do not wish to be included in my named report can be included as confidential comments to the editors, which will not be published.

I agree to the open peer review policy of the journal

To further support our reviewers, we have joined with Publons, where you can gain additional credit to further highlight your hard work (see: https://publons.com/journal/530/gigascience). On publication of this paper, your review will be automatically added to Publons, you can then choose whether or not to claim your Publons credit. I understand this statement.

Yes Choose an item.
