# Supplementary material for: Improving the annotation of the Heterorhabditis bacteriophora genome
Source: Gigascience. 2018 Apr 2;7(4):giy034. doi: 10.1093/gigascience/giy034 (PMC5906903; doi:10.1093/gigascience/giy034)
Supplement: Reviewer_3_Report_(Original_Submission) -- Katharina Hoff — 12/21/2017 Reviewed [file giy034_reviewer_3_report_(original_submission).pdf]

**Reviewer Report**

**Title: Improving the annotation of the Heterorhabditis bacteriophora genome**

**Version: Original Submission** **Date:** 12/21/2017

**Reviewer name: Katharina Hoff**

**Reviewer Comments to Author:**

Dear authors,

thank you for publishing the re-annotation of Heterorhabditis bacteriophora. It is both interesting for the particular research community dealing with Heterorhabditis bacteriophora, as well as for all research communities dealing with non-model organisms, in general. You demonstrate that the software applied for annotating a species can heavily impact conclusions drawn from a genome annotation project; and that it is worth re-annotating also non-model organisms with state of the art tools.

Below, you find my review, structured according to the Guide for GigaScience reviewers.

1. Is the rationale for collecting and analyzing the data well defined?

Yes.

2. Is it clear how data was collected and curated?

Yes, it is very clear.

3. Is it clear - and was a statement provided - on how data and analyses tools used in the study can be accessed?

For data, it is very clear.

The authors also make an effort to demonstrate tool availability (not their own, but software developed by others) by providing RRIDs. However, in some cases, the provided RRIDs are more confusing than helpful.

RRID:SCR_008419 is given for BLAST v2.6.0+ but the RRID leads to an URL that is not available (and in the past, when it was available, it corresponded to a particular BLAST interface for balsting against Aedes aegypti, an organsim that is not relevant to the manuscript under review). In this case, it would be more helpful to provide e.g. an URL to the download location of BLAST v2.6.0+; or create a new RRID.

RRID:SCR_005622 is given for the RNA-Seq aligner STAR; the RRID leads to an URL for a user/password protected STAR related web application. I strongly assume the authors ran STAR locally, and thus, an URL to the offical STAR website would be more appropriate (https://github.com/alexdobin/STAR/releases), or the creation of a new RRID.

For Rstudio, accidentally, the RRID to STAR web application is provided. Please update to correct RRID or URL.

(No RRID or URL is provided for BRAKER. The URL is available in the referenced manuscript, though, and I believe that is sufficient. However, if journal policy is to always print RRIDs or URLs, you might want to add one of the download URLs. Also, BRAKER1 is the only tool where to do not list the version number (braker.pl

--version).)

4. Are accession numbers given or links provided for data that, as a standard, should be submitted to a community approved public repository?

In principle, yes, some accession numbers were still missing during the review process but will be updated by the authors prior publication.

5. Is the data and software available in the public domain under a Creative Commons license?

Scripts implemented particularly for this publication are avaiable at github, the license is GNU Public License V3. There are differences between licenses, I kindly ask the journal to check whether GPL fulfills the journal's requirements.

6. Are the data sound and well controlled?

Yes.

7. Is the interpretation (Analysis and Discussion) well balanced and supported by the data?

Yes.

8. Are the methods appropriate, well described, and include sufficient details and supporting information to allow others to evaluate and replicate the work?

In principle: yes. However, it might be useful to the community to provide not only references to the particular tool and version, but also the exact command lines that were used in this project. It would be really nice if you added the command lines to some supplementary document. For example, a reader who knows that BRAKER1 software, will assume that braker was called with the option --softmasking when the authors state that it was applied to a softmasked genome. A reader who is less familiar with the software will maybe not know this and might thus not be able to replicate the experiments, exactly.

9. What are the strengths and weaknesses of the methods?

The authors used state of the art methods in a very suitable way.

10. Have the authors followed best-practices in reporting standards?

Yes.

11. Can the writing, organization, tables and figures be improved?

I am not a native speaker of English, myself, but I believe the language is good.

I hope that 1.747 as number of protein coding genes predicted by BRAKER1/soft-masked in Table 2 is a typo, please fix.

12. When revisions are requested.

Minor revisions:

Please correct used software accessiblity references as recommended in point 3.

Please correct typo in Table 2 (point 11).

Discretionary revisions:

Please consider my statement to point 8.

The journal should probably have a look at the license issue (point 5).

13. Are there any ethical or competing interests issues you would like to raise?

No.

I hope you find this review useful.

Kind regards,

Katharina Hoff

**Methods**

Are the methods appropriate to the aims of the study, are they well described, and are necessary controls included? Yes

**Conclusions**

Are the conclusions adequately supported by the data shown? Yes

**Reporting Standards**

Does the manuscript adhere to the journal's guidelines on minimum standards of reporting? Yes

Choose an item.

**Statistics**

Are you able to assess all statistics in the manuscript, including the appropriateness of statistical tests used? There are no statistics in the manuscript.

**Quality of Written English**

Please indicate the quality of language in the manuscript: Acceptable

**Declaration of Competing Interests**

Please complete a declaration of competing interests, considering the following questions:

- Have you in the past five years received reimbursements, fees, funding, or salary from an organisation that may in any way gain or lose financially from the publication of this manuscript, either now or in the future?
- Do you hold any stocks or shares in an organisation that may in any way gain or lose financially from the publication of this manuscript, either now or in the future?
- Do you hold or are you currently applying for any patents relating to the content of the manuscript?
- Have you received reimbursements, fees, funding, or salary from an organization that holds or has applied for patents relating to the content of the manuscript?
- Do you have any other financial competing interests?
- Do you have any non-financial competing interests in relation to this paper?

If you can answer no to all of the above, write 'I declare that I have no competing interests' below. If your reply is yes to any, please give details below.

I declare that I have no competing interests

I agree to the open peer review policy of the journal. I understand that my name will be included on my report to the authors and, if the manuscript is accepted for publication, my named report including any attachments I upload will be posted on the website along with the authors' responses. I agree for my report to be made available under an Open Access Creative Commons CC-BY license (http://creativecommons.org/licenses/by/4.0/). I understand that any comments which I do not wish to be included in my named report can be included as confidential comments to the editors, which will not be published.

I agree to the open peer review policy of the journal

To further support our reviewers, we have joined with Publons, where you can gain additional credit to further highlight your hard work (see: https://publons.com/journal/530/gigascience). On publication of this paper, your review will be automatically added to Publons, you can then choose whether or not to claim your Publons credit. I understand this statement.

Yes Choose an item.
